# Ethnobotanical Documentation of Medicinal Plants Used by the Indigenous *Panay Bukidnon* in Lambunao, Iloilo, Philippines

**DOI:** 10.3389/fphar.2021.790567

**Published:** 2022-01-10

**Authors:** Cecilia Salugta Cordero, Ulrich Meve, Grecebio Jonathan Duran Alejandro

**Affiliations:** ^1^ The Graduate School, University of Santo Tomas, Manila, Philippines; ^2^ Biology Department, School of Health Science Professions, St. Dominic College of Asia, City of Bacoor, Philippines; ^3^ Department of Plant Systematics, University of Bayreuth, Bayreuth, Germany; ^4^ College of Science and Research Center for the Natural and Applied Sciences, University of Santo Tomas, Manila, Philippines

**Keywords:** ethnobotany, ethnomedicine, Panay Bukidnon, Panay Island, Philippines

## Abstract

The *Panay Bukidnon* is a group of indigenous peoples living in the interior highlands of Panay Island in Western Visayas, Philippines. Little is known about their ethnobotanical knowledge due to limited written records, and no recent research has been conducted on the medicinal plants they used in ethnomedicine. This study aims to document the medicinal plants used by the indigenous *Panay Bukidnon* in Lambunao, Iloilo, Panay Island. Semi-structured interviews were conducted with 75 key informants from June 2020 to September 2021 to determine the therapeutic use of medicinal plants in traditional medicine. A total of 131 medicinal plant species distributed in 121 genera and 57 families were used to address 91 diseases in 16 different uses or disease categories. The family Fabaceae was best represented with 13 species, followed by Lamiaceae with nine species and Poaceae with eight species. The leaf was the most frequently used plant part and decoction was the most preferred form of preparation. To evaluate the plant importance, use value (UV), relative frequency citation (RFC), relative important index (RI), informant consensus factor (ICF), and fidelity level (FL) were used. *Curcuma longa* L. had the highest UV (0.79), *Artemisia vulgaris* L. had the highest RFC value (0.57), and *Annona muricata* L. had the highest RI value (0.88). Diseases and symptoms or signs involving the respiratory system and injury, poisoning, and certain other consequences of external causes recorded the highest ICF value (0.80). *Blumea balsamifera* (L.) DC. and *Chromolaena odorata* (L.) R.M. King & H. Rob were the most relevant and agreed species for the former and latter disease categories, respectively. *C. odorata* had the highest FL value (100%) and was the most preferred medicinal plant used for cuts and wounds. The results of this study serve as a medium for preserving cultural heritage, ethnopharmacological bases for further drug research and discovery, and preserving biological diversity.

## Introduction

About 370–500 million indigenous peoples live in 90 countries worldwide, making up 5% of the global population. They represent 5,000 distinct and diverse cultures, but they also account for 15% of the extremely poor and deprived communities from social services and economic resources (United Nations Development Programme, 2019).

In the Philippines, there are 110 ethnolinguistic groups with more than 14 million people spread across the archipelago, with the highest population in Mindanao (63%), followed by Luzon (32%) and Visayas (3%) (National Commission on Indigenous Peoples, 2011), who occupy around 13 million hectares (ha) (45%) of the national land territory (National Economic Development Agency, 2017). The *Bukidnon* is the major indigenous group in the Central and Western Visayas in terms of population size, followed by the *Ati/Ata* (Negritoes). In Panay Island in Western Visayas, the *Bukidnon* population is about 112,000. The province of Iloilo is one of the four provinces in Panay Island, together with Aklan, Antique, and Capiz. It has the highest *Bukidnon* population with 62,245 individuals (National Commission on Indigenous Peoples, 2019). *Bukidnon,* which literally translates to the “mountain people,” were once coastal dwellers, but due to the piratical raids from Mindanao and political suppressions during the reign of the Spanish government, they moved to the hinterlands of the island (Magos, 2004). This was depicted in their epic tradition of chanting called *Sugidanon* (Magos, 1999). To distinguish the *Bukidnon* in Panay from the other *Bukidnon* groups in Mindanao, Negros, and other neighboring islands, the “*Panay*” is added (Gowey, 2016). Other authors used *Mundo* (Beyer, 1917), *Monteses* “mountaineer” (Ealdama, 1938), *Sulod* or *Sulodnon* “enclosed by the mountains” (Jocano, 1968; German, 2010), *Tumandok* “native of the place” (Talledo, 2004), and *Bukidnon* (Smith, 1915; Magos, 1999) to designate the *Panay Bukidnon* people.

The *Panay Bukidnon* primarily utilized the forest resources, rivers, and streams for their food and livelihood. They also engaged in slash and burn farming and building boats to transport their goods to the lowlands. In the 1970s, when logging activities were prohibited by the Philippine government, they shifted to other means of living, including farming various crops (Magos, 1999). Their social organization is relatively similar to the lowlanders. Their community membership pattern is composed of the *baylan/babaylan, mirku* (herb doctor), *parangkutun* (advisor), and the *husay* (arbiter). The *baylan* is considered the most important status in society and regarded with high respect. The *baylan* is the one who communicates with the spiritual world, interprets dreams, and handles religious performances. He or she may also administer herb medicine to the sick and practice folk medicine and physical therapy. Their language is *Kiniray-a*, a dialect that is similar to Ilonggo/Hiligaynon. Today the *Panay Bukidnon* settled in the interior “barangays” (villages) of at least 24 municipalities of the four provinces of Panay (Provincial Planning Development Office, 2018; National Commission on Indigenous Peoples, 2020) and most of their communities are located in the mountainous areas of the Central Panay Mountain Range.

Iloilo province is situated in the southeastern part of Panay Island in Western Visayas. It is geographically located at the center of the archipelago, and it is known as the “Heart of the Philippines.” Its excellent port facility and strategic location made the province the center of trade during the 1890s when the sugar industry was booming and it was once given the title of “Queen City of the South.” It is also known for the “*Dinagyang Festival,*” one of the most spectacular religious and cultural celebrations in the country in honor of Senior Sto. Nino (Child Jesus) (Province of Iloilo, 2015). The province has a total land area of 491,940 ha, 24% of which is classified as forestland, while 76% is classified as alienable and disposable land (Department of Environment and Natural Resources, 2019).

Several ethnobotanical surveys in Panay Island have been conducted on the *Ati* (Negritoes) indigenous groups (Madulid et al., 1989; Ong and Kim, 2015; Cordero et al., 2020; Cordero and Alejandro, 2021), but there is no study focused exhaustively on the medicinal plants used by the *Panay Bukidnon* in ethnomedicine. Nevertheless, several plants were listed with medicinal purposes in the anthropological case studies documented in the interior barangays of Tapaz, Capiz in Central Panay in 1945–1959 (Jocano, 1968). Given the absence of recent research about the ethnobotanical knowledge of *Panay Bukidnon*, it is therefore urgent to document this indigenous knowledge before it is forgotten. The documentation of traditional knowledge will serve as a medium for preserving cultural heritage, ethnopharmacological bases of drug research and discovery, and preserving biological diversity. Thus, this study is the first attempt to extensively survey the ethnobotanical knowledge in one of the indigenous *Panay Bukidnon* communities in the province of Iloilo in Panay Island, Western Visayas, Philippines.

## Materials and Methods

### Study Area and Permits

**FIGURE 1:**
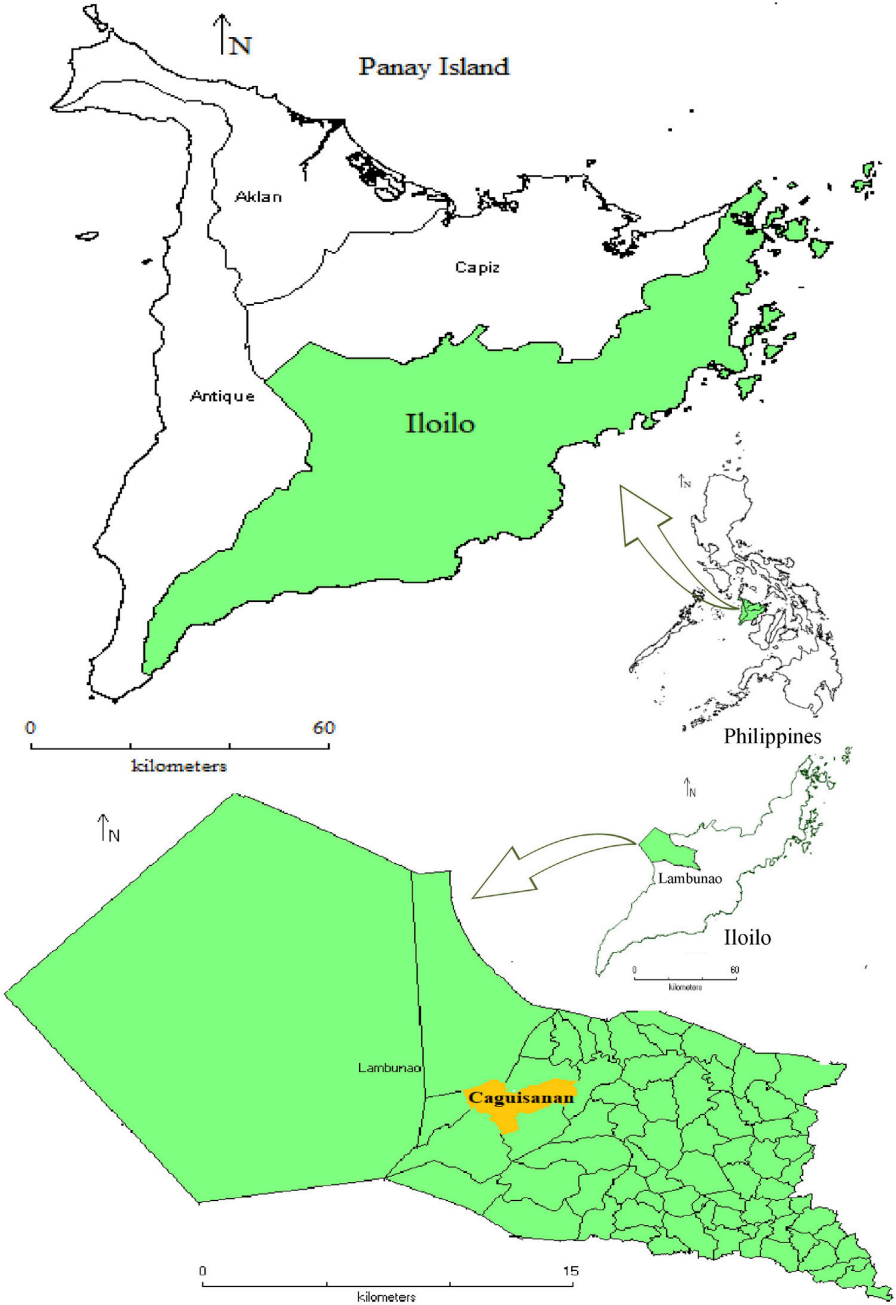
Position of the study area in Panay Island (Philippines).

The town of Lambunao is a first-class municipality in the third district of Iloilo province, with a population of 81,236 individuals as of May 2020 (Philippine Statistics Authority, 2021). It is the largest municipality in the province in terms of land area (40, 709 ha), about 26.12% of which are forestlands, and the rest are alienable and disposable land. It is bounded by the municipalities of Calinog in the North, Duenas and Pototan in the East, Janiuay and Badiangan in the South, and Janiuay and Valderrama, Antique, in the west (Figure 1). It is a mountainous municipality and has the highest elevation (194 m a.s.l.) in the province. The climate of the area has two pronounced seasons: dry from the months of November to April and wet for the rest of the year. Seven out of its 73 barangays are inhabited by the *Panay Bukidnon* people. Brgy. Caguisanan, which lies between 11°05′37.6″N and 11°04′42.3″N latitude and 122°24′26.6″E 122°26′51.6″E longitude, has a land area of about 5.20 km^2^. According to the recent survey, it is one of the seven indigenous *Panay Bukidnon* barangays with a population of 1,842 in 394 households. The main source of livelihood in the barangay is farming of various crops such as rice, banana, corn, and other vegetables. Some of the younger generations are professionals working in various private and government sectors and some are working as overseas Filipino workers abroad. The landscape of the study site is dominated by hills and mountains with scattered rice terraces, grasslands, and patch forests.

Certification Precondition was acquired from the National Commission on Indigenous Peoples (NCIP)-Region VI/VII and the researchers have satisfactorily complied with the requirements for securing the Indigenous Knowledge and Systems Practices (IKSPs) and Customary Laws (CLs). It was issued in compliance with Section 59 of the Republic Act No. 8371 “The Indigenous Peoples Rights Act (IPRA) of 1997.” Several meetings were conducted: Pre-FPIC (Free and Prior Informed Consent) Conference; Disclosure Conference with the Indigenous Peoples (IP) Community and Presentation of Application; Community Decision Meeting; Memorandum of Agreement Preparation and Signing; and Output Validation Meeting. A Wildlife Gratuitous Permit was issued by the Department of Natural Resources Region (DENR) VI before conducting the study.

### Data Collection

Fieldworks and interviews were conducted from June 2020 to September 2021. Interviews were carried out using semi-structured questionnaires, ethically reviewed, and approved (Supplementary Material S1). A purposive sampling technique was used, and the principal key informants were determined during the community decision meeting in the presence of the NCIP officers, barangay officials, IP leader, and council of elders. The informants were composed of the tribal leader, council of elders, herb doctors (*mirku/surhano/albularyo*), midwife (*paltera*), and other members of the community who have indigenous knowledge of using medicinal plants in treating and addressing health problems and conditions. A total of 75 informants, 31 males and 44 females, aged between 24 and 89 years old, were interviewed at their own convenience in their community during the study. Questions regarding personal information and the medicinal plants they used when they experienced any health-related problems were asked during the surveys. The information about the demographic profile of the participants, such as age, gender, civil status, educational attainment, and occupation, is shown in Table 1. The plant part used, mode of preparation, and administration were also recorded during the interviews. A focus group discussion was conducted with the 10 members of the council of elders to verify the acquired data among the informants during the output validation meeting. The meeting was facilitated by the NCIP officers, IP leader, and Brgy. Captain.

**TABLE 1:** Demographic profile of the *Panay Bukidnon* informants in Lambunao, Iloilo, Philippines.

Social group	Variables	No. of informants (75)	Percentage (%)
Sex	Male	31	41
	Female	44	59
Age	24–39	9	12
	40–55	29	39
	56–70	25	33
	71–89	12	16
Civil Status	Married	71	95
	Single	2	3
	Widowed	2	3
Education	No formal education	5	7
	Elementary	37	49
	Secondary	16	21
	Tertiary	17	23
Occupation	Farmer	19	25
	Housewife	29	39
	Self-employed	11	15
	Employed	5	7
	Barangay officials	7	9
	*Albularyo* (herb doctor)	2	3
	*Paltera* (midwife)	1	1
	IP leader	1	1

### Plant Collection and Identification

Collecting medicinal plant samples was carried out with the help of the informants, if available in their immediate surroundings or their home gardens right after the interview. Some field collections were assisted by the informants who have the knowledge of the location of some plants that were not available in the home gardens. Plants were also photographed for documentation purposes. Voucher specimens were prepared using three to five branches with preferably reproductive parts (flowers and fruits), inserted in newspapers, and positioned in a way that best represents the plant in the wild. The plants were poisoned with a generous amount of denatured alcohol in polyethylene bags. Poisoned specimens were then transferred to a new newspaper and placed in a presser. Pressed and dried plant specimens were then mounted on herbarium sheets with proper documentation labels. Voucher specimens were accessioned and deposited in the Herbarium of the Northwestern University Luzon (HNUL). Identification of the collected medicinal plants was made using different online databases such as Co’s Digital Flora of the Philippines, (https://www.philippineplants.org/), Phytoimages (http://www.phytoimages.siu.edu), Stuartxchange (http://www.stuartxchange.org/), and Plants of the World Online (http://plantsoftheworldonline.org/), then verified by Mr. Danilo Tandang, a botanist at the Philippine National Museum Herbarium and Mr. Michael Calaramo of the Herbarium of Northwestern University Luzon (HNUL). For the validation of the family and scientific names, Tropicos (Tropicos, 2021), World Flora Online (World Flora Online, 2021), and International Plant Names Index (International Plant Names Index, 2021) were used. To identify the geographical distribution and endemicity of the medicinal plants, Co’s Digital Flora of the Philippines (Pelser et al., 2011) and Plants of the World Online (Plants of the World Online, 2021) were used.

### Data Analyses

There were five values used to quantify the plant importance: use value (UV), relative frequency of citation (RFC), relative importance index (RI), informant consensus factor (ICF), and fidelity level (FL). The UV was calculated to determine the relative importance of the medicinal plant species using the following formula: UVs=Ui/N , where U_i_ is the number of use reports cited or mentioned by each informant for a particular species and N is the total number of informants (Phillips and Gentry, 1993). A use report was considered every time an informant cited or mentioned a plant being used for any medical condition or purpose. RFC was used to determine the culturally important medicinal plants using the following formula: RFCs=FCs/N , where FC_s_ is the number of informants who cited or mentioned a plant species (frequency citation) and N is the total number of informants who participated in the study. The values range from 0 to 1, 1 being the highest and indicating that all informants cited or mentioned a particular plant species (Tardío and Pardo-De-Santayana, 2008). RI was used to assess the relative importance of medicinal plants by use category using the following formula: RIs=[RFCs(max)+RNUs(max)]/2 , where RFC_s(max)_ (RFC_s(max)_ = FC_s_/FC_max_) is the relative frequency of citation of the species and is obtained by dividing the frequency citation of informant/s for a particular species (FC_s_) by the number of informants citation of the species that has the maximum or highest frequency citation (FC_max_). RNU_s(max)_, (RN_s(max)_ = NU_s_/NU_max_) is the relative number of the use categories and is obtained by dividing the number of use categories of a particular species (NU_S_) by the number of use categories of the species with the highest use categories (NU_max_). Values closest to 1 indicate that the medicinal plants are most frequently cited as useful in different use categories (Tardío and Pardo-De-Santayana, 2008). ICF was used to evaluate the consensus or homogeneity of the ethnobotanical information from the participating informants using the following formula: ICF=(Nur−Nt)/(Nur−1) **,** where N_ur_ is the number of use reports for each disease category and N_t_ is the number of species used in that category (Heinrich et al., 1998). FL was used to assess the percentage of the most preferred medicinal plant for a particular disease category using the following formula: FL=(Np/N) x100 , where N_p_ is the number of informants who cited or mentioned the use of a medicinal plant for a particular disease category and N is the total number of informants who cited that plant for any other use or purpose (Friedman et al., 1986). A high value indicates that a medicinal plant has the highest use report and the most preferred species within a particular disease category. There were 16 different use or disease categories adapted and modified from the ICD-11 International Classification of Diseases 11th Revision of the World Health Organization (World Health Organization, 2021), which is used in this ethnopharmacological documentation.

## Results

### Medicinal Plants Characteristics

**FIGURE 2:**
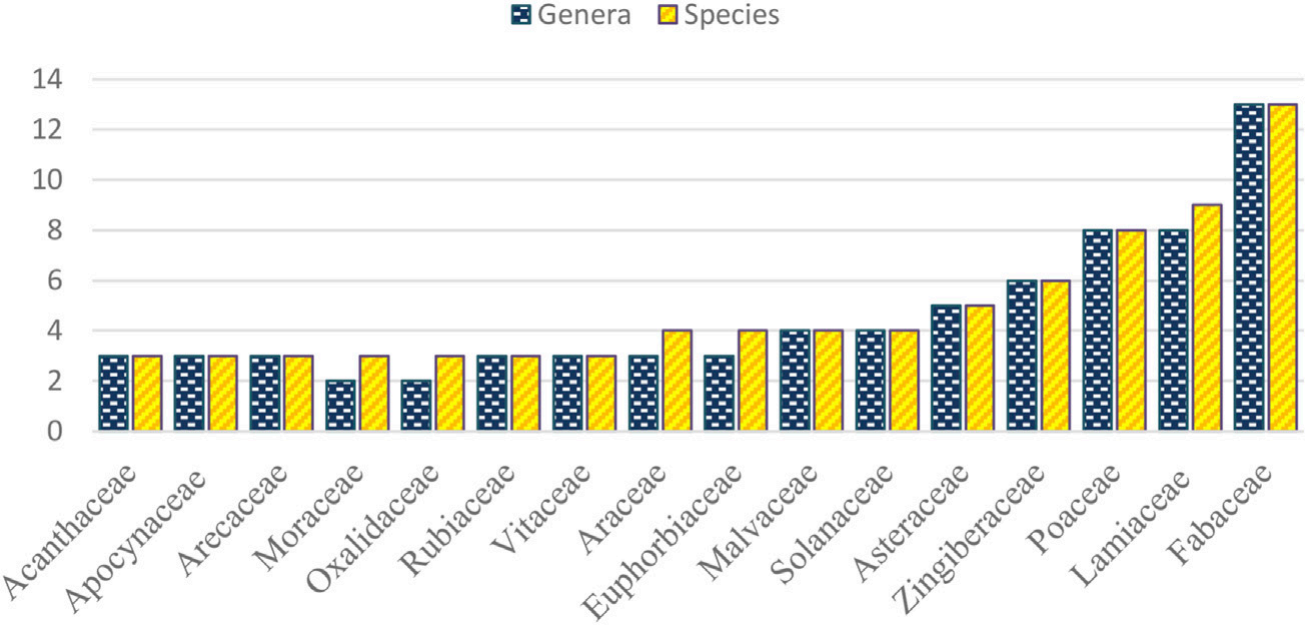
Family of medicinal plants with a high number of genera and species.

The present study documented a total of 131 medicinal plant species distributed in 121 genera and 57 families. The family Fabaceae was best represented with 13 species, followed by Lamiaceae with nine species and Poaceae with eight species (Figure 2). Fabaceae are used to treat 28 diseases in 13 different use or disease categories, Lamiaceae in 24 diseases in ten disease categories, and Poaceae in 21 diseases in 12 disease categories.

**FIGURE 3:**
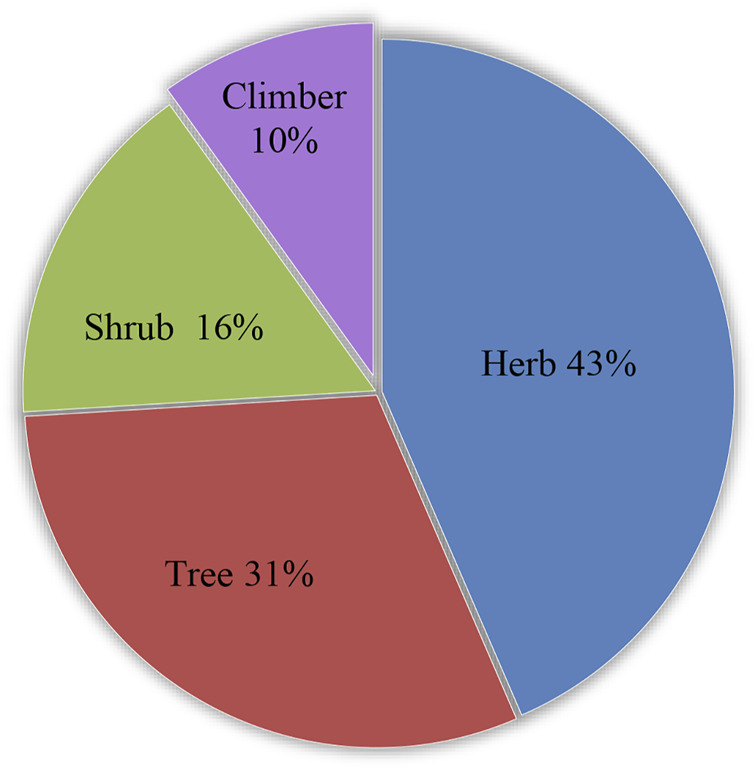
Medicinal plant growth habit.

The medicinal plants recorded possess different growth forms such as herbs (43%), trees (31%), shrubs (16%), and climbers (10%) (Figure 3). The plants were collected within the vicinity of the barangay mostly cultivated in the informant’s home gardens or backyards that serve as ornamentals and vegetables and used for medicinal purposes; some were cultivated as crops in the farmland; some were grown on the riverbanks and forest; others do grow as weeds pervasively around the community. Of all the 131 medicinal plants listed, 91 species were collected as cultivated plants and 40 species were collected in the wild. Out of 127 plants identified up to the species level, 78 species are not native (introduced, naturalized, cultivated) in the Philippines and 49 species are native. Three species (*Areca catechu* L., *Musa textilis* Née, and *Mussaenda philippica* A. Rich.) of the native plants are considered endemic and their occurrence is widespread in the country. Information about the plant growth habit, collection sites, and geographical distribution and endemicity are found in = Supplementary Table S1.

The medicinal plant details are summarized in Supplementary Table S2. The scientific, local, and family names are also listed along with the plant part used, disease or purpose, quantity, mode of preparation, the form of administration, adverse or side effects, use value, relative frequency citation, and relative importance index.

### Plant Part Used and Mode of Preparation and Administration

**FIGURE 4:**
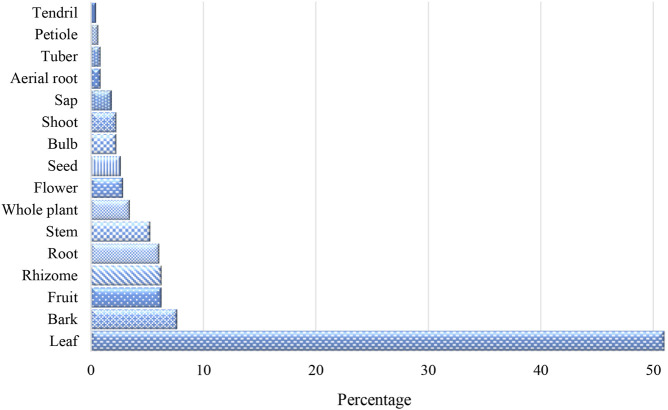
Medicinal plant parts used.

**FIGURE 5:**
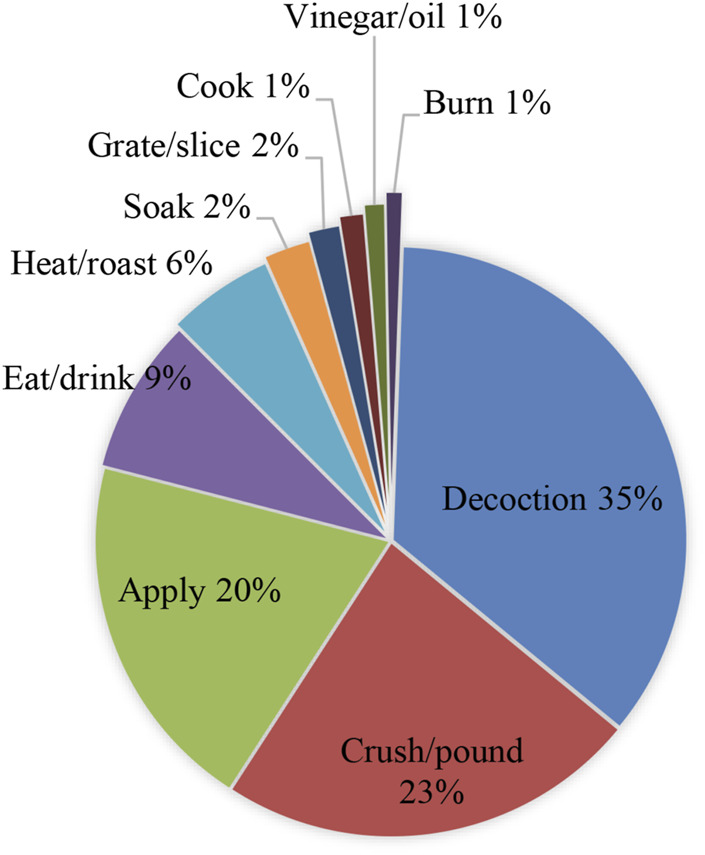
Medicinal plant preparation.

Sixteen different medicinal plant parts were used to address 94 diseases and health-related conditions documented in this study. The most frequently used plant parts for the preparation of the remedy were leaf (51%), followed by bark (8%), fruit (6%), and rhizome (6%) (Figure 4). Root, stem, whole plant, flower, seed, bulb, shoot, sap, and aerial root were also used but less frequently. The least utilized plant parts were tuber, petiole, and tendril. There were ten different ways to prepare the medicinal plants and the most common forms were decoction (35%), followed by crushing or pounding (23%) and direct application (20%) (Figure 5). Eat/chew/drink, heat/roast, soak in water, and grate/slice were also practiced. The least forms of preparation were cooking, processing into vinegar or oil, and burning for smoke or ash. The plant parts used and the mode of preparation of the medicinal plants depend on the ailments to be addressed and to whom they will be administered. Occasionally, some of the preparations include animal parts and products such as blood, egg, beeswax (*kabulay*), slaked lime (*apog*), minerals like salt, and chemicals like kerosene but in minute amounts. Sugar or breastmilk was also added to reduce or mask the bitterness of plant extracts to be taken orally by infants and children.

More than half of the medicinal plant preparations (52%) recorded were administered externally or topically by applying plant parts directly on the body, rubbing plant extracts, bathing, and burning for smoke and ash. The rest were taken orally (48%) by drinking decoction, eating, chewing, drinking extracts or liquids and used as a mouthwash.

### Quantity and Dosage

The quantity of the medicinal plants used is influenced by the guided cultural and religious beliefs of the *Panay Bukidnon* and should be prepared or administered in odd numbers (3, 5, or 7). For example, in treating headaches, three different medicinal plants such as *Pseuderanthemum carruthersii* (Seem.) Guillaumin (3, 5, or 7 leaves depending on the leaf size), *Curcuma longa* L. (7 thinly sliced rhizomes), and *Zingiber officinale* Roscoe (7 thinly sliced rhizomes) were applied on the forehead. The frequency of the administration was dependent on the disease to be treated. For decoction, it was often administered by drinking a full glass to be taken twice or thrice a day or as a replacement for water intake. The detailed quantity and frequency of administration of medicinal plants are shown in Supplementary Table S2.

### Use Value and Relative Frequency of Citation

The use value was used to evaluate the relative importance of the medicinal plants: high values indicate high use report, while relative frequency citation determined the usefulness of the plant by high FC or being mentioned by all the informants.

The top three medicinal plants with the highest use value were *Curcuma longa* L. (0.79), *Blumea balsamifera* (L.) DC. (0.64), and *Artemisia vulgaris* L. (0.59) (Table 2). *C. longa* is used to treat 13 diseases in nine disease categories and recorded a high use report in suppressing fever, headache, and *sinda.* It is usually prepared with two or four medicinal plants. The preparation and mode of administration for headache and fever were the same and with few modifications for *sinda*. *C. longa* is also used for muscle pain, stomachache, bloated stomach, tooth decay, typhus, typhoid fever, memory loss, cancer, cuts/wounds, and tetanus. It is cultivated in the informant’s home gardens for medicinal purposes.

**TABLE 2:** Top ten medicinal plants with the highest UV, RFC, and RI values.

Rank	Medicinal plants	UV	Medicinal plants	RFC	Medicinal plants	RI
1	*Curcuma longa*	0.79	*Artemisia vulgaris*	0.57	*Annona muricata*	0.88
2	*Blumea balsamifera*	0.64	*Curcuma longa*	0.47	*Curcuma longa*	0.87
3	*Artemisia vulgaris*	0.59	*Blumea balsamifera*	0.44	*B lumea balsamifera*	0.80
4	*Annona muricata*	0.56	*Zingiber officinale*	0.43	*Zingiber officinale*	0.78
5	*Jatropha curcas*	0.55	*Psidium guajava*	0.40	*Artemisia vulgaris*	0.77
6	*Psidium guajava* L	0.55	*Pseuderanthemum carruthersii*	0.40	*Jatropha curcas*	0.72
7	*Justicia gendarussa*	0.52	*Musa balbisiana* cv	0.40	*Moringa oleifera*	0.72
8	*Pseuderanthemum carruthersii*	0.52	*Plectranthus scutellarioides*	0.40	*Plectranthus scutellarioides*	0.62
9	*Musa balbisiana* cv	0.52	*Annona muricata*	0.39	*Psidium guajava*	0.62
10	*Plectranthus scutellarioides/Zingiber offinale*	0.49	*Justicia gendarussa*	0.37	*Carica papaya/Justicia gendarussa*	0.60

*B. balsamifera* was used to treat nine conditions or purposes under eight different disease categories and is widely known to cure cough, used in postpartum care and recovery, and relieved headache. It is also used for muscle pain, bloated stomach/gas pain, goiter, urinary tract infection (UTI), vomiting blood, and *inaswang*. It was collected growing in the farmland, but some informants also cultivated it in their backyards.

*A. vulgaris* was used to treat seven ailments in six disease categories and is the best-known therapy for cough, fever, headache, and body pains. It is also used for the remedy of chest pain, fracture, and hearing problems. It is grown in the home gardens as medicinal plants for future use. Seven medicinal plants were recorded with only one use report and FC for each species: *Cheilocostus speciosus* (J.Koenig) C.D.Specht*, Luffa aegyptiaca* Mill., *Syzygium cumini* (L.) Skeels*, Lygodium circinnatum* (Burm. f.) Sw*.*, *Solanum melongena* L*., Nauclea orientalis* (L.) L*.,* and *Leea guineensis* G. Don, which garnered the lowest value for UV (0.01) and RFC (0.01).

Medicinal plants with the highest RFC were *Artemisia vulgaris* (0.57), followed by *Curcuma longa* (0.47) and *Blumea balsamifera* (0.44) (Table 2). Out of the 75 informants who participated in the survey, *A. vulgaris* had the highest informant citation or FC.

### Relative Importance Index

The RI was used to assess the relative importance of the medicinal plants by use or disease categories. A high value indicates that a particular medicinal plant species is most frequently cited as useful with a high number of use categories or having multiple uses. The top three plants with the highest RI values were *Annona muricata* L. (0.88), *C. longa* (0.87), and *B. balsamifera* (0.80) (Table 2). *A. muricata* is used in 11 different use or disease categories: diseases of the genitourinary system; neoplasms; diseases of the circulatory system and blood or blood-forming organs; endocrine, nutritional and metabolic diseases; diseases and symptoms or signs involving the respiratory system; diseases and symptoms or signs involving the nervous system; diseases and symptoms or signs involving the digestive system or abdomen; infectious and parasitic diseases; diseases and symptoms or signs involving the skin; diseases and symptoms or signs of the musculoskeletal system or connective tissue; and other cultural uses. It is used to address 13 diseases or purposes and recorded the high use report in treating UTI, cancer, and hypertension by drinking the leaf decoction or soaking the young leaves in warm water and drink or by just eating a medium sliced fruit three times a day. *A. muricata* is also used for the remedy of high uric acid, pneumonia, dizziness, intestinal cleansing, kidney trouble, itchy throat, amoebiasis, lump, arthritis, and *doklong*. *C. longa* was used in nine categories: injury, poisoning and certain other consequences of external causes; neoplasms; diseases and symptoms or signs involving the nervous system; general symptoms and signs; diseases and symptoms or signs involving the digestive system or abdomen; infectious and parasitic diseases; diseases and symptoms or signs of the musculoskeletal system or connective tissue; mental or behavioral symptoms, signs, or clinical findings; other cultural uses. *B. balsamifera* is used in eight disease categories: diseases and symptoms or signs involving the digestive system or abdomen; endocrine, nutritional, and metabolic diseases; diseases of the genitourinary system; diseases and symptoms or signs of the musculoskeletal system or connective tissue; diseases and symptoms or signs involving the nervous system; diseases and symptoms or signs involving the respiratory system; pregnancy, childbirth, and the puerperium; other cultural uses. The top ten medicinal plants with the highest use value, relative frequency citation, and relative importance index are shown in Table 2.

### Informant Consensus Factor

There were 91 diseases and purposes in 16 different use or disease categories recorded in this study (Table 3). ICF was used to evaluate the consensus in the medicinal plant information among the informants. High ICF values indicate one or few medicinal plants mentioned by a high number of informants within a particular disease category, and low values indicate that more species are being used and the informants differ in their preference on which plant to use. The highest ICF value (0.80) was in the diseases and symptoms or signs involving the respiratory system and in injury, poisoning, and certain other consequences of external causes. The diseases and symptoms or signs involving the respiratory system were cough, pneumonia, rhinorrhea, itchy throat, chest pain, and tonsillitis. *B. balsamifera* had the highest use report within the category and was frequently used plant for treating cough by consuming the young leaves or by drinking leaf or root decoction or by rubbing leaf extract on the head of the afflicted ones. A high proportion of informants mentioned and agreed upon the use of *B. balsamifera* in treating cough within the category. Injuries, poisoning, and certain other consequences of external causes recorded, such as animal bite, bruise, burn, caterpillar dermatitis, circumcision, cuts and wounds, fracture, and splinter, were the reported purposes or medical uses. *Chromolaena odorata* (L.) R.M.King & H. Rob*.* had the highest use report within the category and was the most preferred medicinal plant used to treat cuts/wounds by applying crushed leaves on the affected area. The next highest value was in the diseases and symptoms or signs involving the nervous system (0.73) with headache, migraine, and dizziness as the reported medical condition. *Pseuderanthemum carruthersii* had the highest use report and was widely used for treating headaches by applying leaves on the forehead alone or with *C. longa* and *Z. officinale*. The lowest ICF value was recorded in mental disorder (0.00) with memory loss as the reported condition and *Curcuma longa* was used for the treatment.

### Fidelity Level

The FL was used to determine the relative importance of a medicinal plant within each category. Medicinal plants with the highest FL values were *Chromolaena odorata* (100%), *Bambusa spinosa* Roxb. (93%), and *Pseuderanthemum carruthersii* (93%) (Table 3). *C. odorata* is exclusively used to treat cuts and wounds and can be seen growing invasively along the paths and roadsides in the community. *B. spinosa* recorded the highest use report for postpartum care and recovery under the pregnancy, childbirth, and puerperium category. It is preferably used and highly suggested by many informants for postpartum care and recovery therapy. Decoction of at least three up to ten different medicinal plants was used for drinking (1–2 glasses), body steaming, and bathing to be performed nine days after a mother gave birth. *B. spinosa* has also been used to treat cancer, UTI, and kidney stones but with only one citation recorded for each ailment *P. carruthersii* has the highest use report and is the most preferred medicinal plant for relieving headaches under the diseases and symptoms or signs involving the nervous system. The lowest FL value was recorded for *C. longa* in treating memory loss under the mental disorder category with only one informant mentioned for its curative effect.

**TABLE 3:** Use or disease category, reported disease or uses, ICF, and FL of the most cited species.

Use or disease- category	Reported diseases or uses under each category*	No. of used taxa	Use report	ICF	Most cited species for each category	Fidelity level (%)	Use or purpose of the most cited taxa
Infectious and parasitic diseases	Amoebiasis (4), athletes foot (1), boil (11), chicken pox (2), dengue (2), helminthiases (2), measles (3), mumps (6), oral thrush (1), shingles (3), ringworm (2), tetanus (2) Pityriasis versicolor (2), tuberculosis (3), typhus (2), typhoid fever (11), wart (2)	59	153	0.62	*Carica papaya*	60	Typhoid fever
Neoplasms	Cancer (12)	12	27	0.58	*Annona muricata*	31	Cancer
Endocrine, nutritional and metabolic diseases	Diabetes (5), goiter (1), high uric acid (1)	7	8	0.14	*Morinda citrifolia*	15	Diabetes
Diseases and symptoms or signs involving the nervous system	Headache (35), migraine (1), dizziness (8), cramps/spasm (4)	48	178	0.73	*Pseuderanthemum carruthersii*	93	Headache
Diseases of the ear or mastoid process, visual system, and symptoms or signs involving speech or voice	Hearing impairment (2), conjunctivitis (1), hoarseness (2)	5	9	0.50	*Moringa oleifera*	15	Conjunctivitis
Diseases of the circulatory system and blood or blood-forming organs	Anemia (6), hypertension (11)	17	28	0.41	*Annona muricata*	24	Hypertension
Diseases and symptoms or signs involving the respiratory system	Cough (24), pneumonia (1), rhinorrhea (2), itchy throat (1), chest pain (1), tonsilitis (1)	30	148	0.80	*Blumea balsamifera*	91	Cough
Diseases and symptoms or signs involving the digestive system or abdomen	Abdominal pain (2), angular cheilitis (1), bleeding gums (1), bloated stomach/gas pain (17), blood in feces (1), constipation (2), diarrhea (17), gastric ulcer (1), halitosis (1), indigestion (1) intestinal cleansing (1), nausea (1), stomachache (39), teething syndrome (1), tooth decay (11) vomiting blood (7)	103	252	0.59	*Chrysophyllum cainito*	67	Diarrhea
Diseases and symptom or signs involving the skin	Dandruff (1), eczema (1), lump (5), Pityriasis rosea (2), rashes (2), skin lesion (5)	16	35	0.56	*Psidium guajava*	20	Skin lesion
Diseases and symptoms or signs of the musculoskeletal system or connective tissue	Lower back pain (2), limb pain (3), muscle pain (8), muscle swelling (2), rheumatoid arthritis (8)	22	51	0.58	*Artemisia vulgaris*	26	Muscle pain
Diseases of the genitourinary system	Induce period/menstruation (1), kidney stones (7), kidney trouble (13), swelling of male genitalia (2), urinary tract infection (22)	45	92	0.52	*Annona muricata*	55	UTI
Pregnancy, childbirth and the puerperium	Breast engorgement (3), birth control (4), induce labor (2), lactation support (1), postpartum bleeding (2), postpartum care and recovery (22)	34	118	0.72	*Bambusa spinosa*	93	Postpartum care and recovery
Injury, poisoning and certain other consequences of external causes	Animal bite (2), bruise (1), burn (1), caterpillar dermatitis (1), circumcision (1), cuts and wounds (21), fracture (7), splinter (2)	36	176	0.80	*Chromolaena odorata*	100	Cuts and wounds
General symptoms and signs	Chill (2), fever (19), malaise (1)	21	59	0.66	*Curcuma longa*	43	Fever
Mental or behavioral symptoms, signs or clinical findings	Memory loss	1	1		*Curcuma longa*	2	Memory loss
Other cultural uses	*Doklong* (14), *hiwit* (2), *inaswang* (5), *kolebra* (3), *sinda* (10)	34	89	0.63	*Curcuma longa*	46	*Sinda*

*Number inside the parenthesis denotes the number of species in each reported disease or use.

### Comparing Different Indices

Table 2 shows the top 10 medicinal plants with the highest UV, RFC, and RI values. High UV and RFC values indicate the high number of use reports and frequency citations (FC) from the informants, while high RI values consider the multiplicity of uses or the high number of uses in different disease categories. This implies that medicinal plants with high UV, RFC, and RI values are the most important and valued medicinal plants in the community. There are a few considerable differences in species ranking yielded by the three indices set out in Table 2. The ranks of the first three species (*C. longa, Blumea balsamifera,* and *Artemisia vulgaris*) are nearly the same in all indices except in RI where *Annona muricata* had the highest value but ranked 4th in UV and only 9th in RFC. *C. longa, B. balsamifera,* and *A. vulgaris* had the highest use reports and FC from the informants and only next to *A. muricata* in terms of multiple uses in different disease categories. *A. muricata* had the highest number of uses or purposes in different disease categories (11 disease categories); however, its use reports and FC are not quite as high as those of the first three species mentioned above. *A. muricata* is the most frequently used medicinal plant in a wide range of diseases (13 diseases). Another noticeable difference is the inclusion of *Moringa oleifera Lam.* and *Carica papaya* L. in the RI index, which are not shown in the top ten species with high UV and RFC values. *M. oleifera* ranks 12th in UV and 13th in RFC, while *C. papaya* ranks 17th in UV and 14th in RFC (values are not shown in Table 2 but available in Supplementary Table S2). Their UV and RFC values are not quite as high, but they have multiple uses in different disease categories. Low UV, RFC, and RI values indicate a low number of use reports, FC, and have one or few uses in disease categories. For example, *Andrographis paniculata* (Burm.f.) has low UV (0.04), RFC (0.03), and RI (0.07) values, indicates low use report (2) and FC (2 informants), and is only used in one disease category (diseases and symptoms or signs involving the digestive system or abdomen). This implies that *A. paniculata* is a less important and less preferred medicinal plant species in the *Panay Bukidnon* community. On the other hand, Table 3 shows the use or disease categories and ICF and FL values. High ICF values are considered the most culturally relevant medicinal plants and the agreement of its use within a disease category in the community, while FL highlights the most preferred species for a particular disease. Medicinal plants with the high ICF values were *Blumea balsamifera* (0.80), *Chromolaena odorata* (0.80), and *Pseuderanthemum carruthersii* (0.73) and these species are highly agreed upon by most informants for the therapy of diseases in their respective disease categories. Medicinal plants with high FL values were *C. odorata* (100%), *P. carruthersii* (93%), and *Bambusa spinosa* (93%) and these are the most preferred species to a particular disease in each disease category. Though *B. spinosa* is not included in the top medicinal plants with high UV, RFC, and RI values, its curative effect for postpartum care and recovery is preferred by a high proportion of informants. Medicinal plants with high UV, RFC, RI, ICF, and FL values are the most culturally important, relevant, preferred, and agreed on species in the *Panay Bukidnon* communities.

### Cultural Important Medicinal Plants

Indigenous peoples are strongly tied with their spiritual beliefs and practices. Interestingly, up to date, the *Panay Bukidnon* still believe that some of the illnesses and diseases are caused by spirits, supernatural beings, and sorcery. Some diseases were mentioned that were caused by *aswang* (witch), *hiwit* (sorcery), *sinda* (charm of spirits) and some health conditions like *doklong* and *kolebra* with more complicated and sometimes unexplained symptoms. *Sinda* is a condition with symptoms like dizziness and fever caused by spirits or supernatural beings, while *kolebra* has symptoms like chills, stomachache, nausea, shortness of breath, and paleness. *Doklong* is somewhat similar to “relapse” and sometimes accompanied by other symptoms like headache, muscle pain, and weakness. For *inaswang*, five medicinal plants were recorded for the therapy and a cultivar of *Alocasia* is frequently used by applying the heated leaf to the stomach area. For the treatment of *hiwit*, a species of *Amomum* is used by rubbing the stem extract on the body or by crushing the stem with the inner bark of *Pipturus arborescens* (Link) C.B. Rob. and taking the extract orally. *Curcuma longa* is the most used medicinal plant used to cure *sinda* by rubbing the rhizome’s extract on the head or applying the sliced rhizome along with other plants on the forehead. *Jatropha curcas* L. is frequently used as a remedy for *kolebra* by drinking the extract of the inner bark and for *doklong*, drinking the leaf decoction of *Citrus maxima* (Burm.) Merr. alone or with other medicinal plants is the most preferred.

### Medicinal Plants Used to Strengthen Immunity Against Infection and for Potential COVID-19 Therapy

With the current situation of the novel coronavirus disease (COVID-19) in the country and the resurgences of the infection waves, communities in the far-flung areas tend to explore different medical plants as an alternative for potential therapy while waiting for the vaccine. There were ten medicinal plants mentioned by the council of elders that they used to boost their immunity against COVID-19 infection (Table 4). If someone is suspected of having a COVID-19 infection or exhibits symptoms related to COVID-19, they use the available medicinal plants (Table 2) available to alleviate their condition. Medicinal plants such as *Curcuma longa, Zingiber officinale, Capsicum annuum* L., and *Peperomia pellucida* (L.) Kunth are traditionally used by the *Panay Bukidnon* to treat fever, headache, cough, and body pains which were also the symptoms of COVID-19 and influenza. They also believed that chewing betel quid which is composed of *Areca catechu* L., *Piper betle* L., *Nicotiana tabacum* L.*,* and slaked lime (*apog*) can help them fight the infection and help them feel substantially better.

**TABLE 4:** Medicinal plants used to strengthen immunity against infection.

Medicinal plants	Preparation and administration
*Capsicum annuum*	Eat one fruit before dinner
*Vitex trifolia*	Boil handful of leaves and drink decoction like water
*Euphorbia hirta*	Boil handful of whole plant and drink decoction as water intake
*Musa balbisiana* cv	Eat ripe fruits three times a day
*Peperomia pellucida*	Soak a handful of whole plants in warm water then drink like water
*Curcuma longa*	Pound a handful of rhizomes then rub extract on the body three times a day
*Zingiber officinale*	Boil rhizome and drink one glass of decoction three times a day
*Areca catechu*, *Piper betle*, and *Nicotiana tabacum*	Wrap a pinch of *A. catechu* seeds in a *P. betle* leaf daubed with slaked lime (*apog*) and plug cut dried leaf of *N. tabacum* then chew (chewing betle quid)

### Comparative Review of the Medicinal Plants With Other Ethnobotanical Studies

The anthropological study conducted in the *Panay Bukidnon* communities in the Province of Capiz in the 1950s recorded a total of 54 medicinal plant species and 21 of which are cited in this current ethnobotanical study. An additional 109 taxa were documented to the medicinal flora used by the *Panay Bukidnon* in Panay Island from this present study.

To identify the new medicinal plants and plant use, a comprehensive comparison was performed with 22 ethnobotanical studies published from 2011 up to the present and with one online database. Only the scientific names and their synonyms were used for the comparative review; local names were not considered because they are arbitrary within different cultures and dialects. Of the 127 medicinal plants identified up to the species level, three species (*Eleutherine palmifolia* (L.) Merr., *Hibiscus acetosella* Welw. ex Hiern, and *Oxalis triangularis* A.St.-Hil.) show some novel medicinal uses that were not documented in other existing ethnobotanical studies conducted in the country. These medicinal plant species are not native to the Philippines. *E. palmifolia* is used for the therapy of rashes and headaches. Its red bulb is the preferred plant part for the treatment. It is usually used as an ornamental plant grown in the home gardens and community center. *H. acetosella* is used to cure cuts/wounds, boils, anemia, hypertension, diabetes, rhinorrhea, and cough. Filipinos also used this medicinal plant as a vegetable and normally use it to “sour” the dishes. With its deep red-purple foliage, it is also served as an ornamental plant. *O. triangularis* is used to treat cuts/wounds by the *Panay Bukidnon* and serves as a hanging ornamental plant for its striking deep maroon trifoliate leaves. Forty-seven medicinal plants were recorded to have an additional therapeutic use or purpose not mentioned in the other previous ethnobotanical studies, while 80 species have the same medicinal values as mentioned in the existing literature. Some of the additional uses or purposes of the medicinal plants that are rarely listed in other studies are angular cheilitis, breast engorgement, and promoting teething in toddlers. The detailed information about the comparative review of the medicinal plants and the additional plant uses is shown in Table 5.

**TABLE 5:** Comparative presence-absence matrix of the medicinal plants used by the *Panay Bukidnon* with other ethnobotanical studies.

Scientific name	1	2	3	4	5	6	7	8	9	10	11	12	13	14	15	16	17	18	19	20	21	22	23	New medicinal plant uses/purpose
*Andrographis paniculata* (Burm.f.) Nees	0	0	0	0	0	0	1	0	0	0	0	0	1	0	0	1	1	0	1	0	0	1	1	
*Justicia gendarussa* Burm.f.*	0	0	0	0	0	0	1	0	0	1	0	0	0	1	0	1	0	0	1	1	1	0	1	
*Pseuderanthemum carruthersii* (Seem.) Guillaumin	0	0	0	0	0	0	1	0	0	0	0	0	0	0	0	1	0	0	0	1	0	0	1	
*Acorus calamus* L.	1	0	0	0	0	0	0	0	0	0	0	0	0	0	0	0	0	0	0	1	0	0	1	
*Amaranthus viridis* L.	0	0	0	0	0	1	0	0	0	0	0	0	0	0	0	0	0	0	0	0	0	0	0	Anemia
*Alternanthera sessilis* (L.) R. Br. ex DC.	0	0	0	0	0	0	0	0	0	0	0	0	0	0	0	0	0	0	0	0	0	0	0	Anemia, vomiting blood
*Allium sativum* L.	1	1	0	1	1	0	1	0	1	0	0	0	1	1	1	1	0	0	0	0	1	0	1	
*Allium fistulosum* L.	0	0	0	0	0	0	0	0	0	0	0	0	0	0	0	0	0	0	0	0	0	0	0	Teething syndrome
*Mangifera indica* L.	1	1	0	1	1	1	1	0	1	1	0	0	1	1	0	1	1	0	0	0	1	0	1	Stomachache, cough, kidney trouble,
*Spondias pinnata* (L.f.) Kurz	0	0	1	0	0	0	0	0	0	0	0	0	0	0	0	0	1	0	1	0	0	0	0	
*Annona muricata* L.	0	1	1	0	1	0	1	0	1	0	0	1	1	1	1	1	1	0	0	1	1	1	1	Pneumonia, lumps
*Annona squamosa* L.	0	0	1	1	1	1	1	0	1	0	0	1	0	0	0	0	0	0	0	0	1	0	1	Postpartum care and recovery
*Centella asiatica* (L.) Urb	1	0	1	0	0	1	1	1	1	0	0	1	0	0	0	1	0	0	0	0	0	0	1	
*Alstonia scholaris* (L.) R.Br.*	1	0	0	0	0	0	1	0	0	0	0	0	1	0	0	1	0	0	0	1	0	0	1	
*Catharanthus roseus* (L.) G.Don	0	0	1	0	0	0	1	0	1	0	0	0	1	0	0	1	0	0	0	1	0	0	1	
*Tabernaemontana pandacaqui* Poir.*	0	0	1	1	0	0	1	0	0	0	0	0	1	0	0	1	0	0	0	1	0	0	1	
*Alocasia macrorrhizos* (L.) G.Don	0	0	0	0	0	0	1	0	0	1	0	0	0	0	0	0	0	0	0	1	0	0	1	
*Alocasia* cultivar																								
*Colocasia esculenta* (L.) Schott	0	0	1	1	1	0	0	0	0	0	0	0	0	1	0	0	0	0	0	0	0	0	1	Breast engorgement
*Homalomena philippinensis* Engl	0	0	0	0	0	0	0	0	0	1	0	0	0	0	0	1	1	1	0	0	0	0	0	
*Schefflera elliptica* (Blume) Harms	0	0	0	0	0	0	1	0	0	0	0	0	0	0	0	0	0	0	0	0	0	0	0	
*Areca catechu* L.	1	0	1	1	1	0	1	0	1	1	0	0	0	0	0	1	1	0	0	1	0	0	1	
*Cocos nucifera* L.	0	1	1	1	1	1	1	0	1	0	0	1	1	0	0	1	0	0	0	1	0	0	1	
*Corypha utan* Lam.	0	0	0	0	0	0	1	0	0	0	0	0	0	1	0	0	0	0	0	0	0	0	1	
*Cordyline fruticosa* (L.) A.Chev.*	1	0	1	0	0	0	1	0	0	1	0	0	0	1	0	0	0	0	0	0	0	0	1	
*Aloe vera* (L.) Burm.f.	0	0	0	0	0	1	1	0	1	0	0	0	1	1	1	1	0	0	0	0	0	0	1	
*Artemisia vulgaris* L.	1	1	0	0	1	1	1	0	1	0	0	1	1	0	1	1	1	0	1	1	1	1	1	
*Bidens pilosa* L.	1	1	0	0	0	0	0	1	1	0	0	0	0	0	0	0	1	0	0	0	0	0	1	Toothache
*Blumea balsamifera* (L.) DC.*	1	0	1	1	1	1	1	0	1	0	0	1	0	0	0	1	1	1	1	1	1	1	1	
*Chromolaena odorata* (L.) R.M.King and H.Rob	0	1	0	0	1	1	1	0	1	1	0	0	1	1	0	1	1	1	1	1	1	0	1	
*Elephantopus tomentosus* L.*	1	0	0	0	0	0	0	0	1	0	0	0	0	0	0	1	0	1	0	1	0	0	1	
*Impatiens balsamina* L.	0	0	1	1	0	1	1	0	0	0	0	0	1	0	1	0	0	0	1	0	1	0	1	
*Basella alba* L.	0	0	0	0	0	0	0	0	0	0	0	0	0	1	0	0	0	0	0	0	0	0	1	Mumps
*Bixa orellana* L.	0	0	1	0	1	1	1	0	1	0	0	0	1	0	1	0	0	0	0	0	1	0	1	Bloated stomach/gas pain
*Cordia dichotoma* G.Forst.	0	1	0	0	1	0	0	0	0	1	0	0	1	0	0	0	0	0	1	1	0	0	1	
*Brassica rapa* L.	0	0	0	0	0	0	0	0	0	0	0	0	0	0	0	0	0	0	0	0	0	0	0	Kidney problem, anemia
*Ananas comosus* (L.) Merr.	1	0	0	1	1	0	1	0	1	0	0	1	1	0	0	1	1	0	0	1	0	0	1	Bleeding gums
*Carica papay*a L.	1	1	1	1	1	1	1	0	1	0	0	0	1	1	1	1	1	0	1	1	1	0	1	
*Ipomoea batatas* (L.) Lam	0	0	1	1	1	0	1	0	0	0	0	0	1	1	0	0	0	0	0	0	0	0	1	Breast engorgement
*Decalobanthus peltatus* (L.) A.R.Simões and Staples*	0	0	0	0	0	0	0	0	0	0	0	0	0	0	0	1	0	0	0	0	0	0	1	
*Cheilocostus speciosus* (J.Koenig) C.D.Specht	0	0	0	0	0	0	0	0	0	0	0	0	0	0	0	1	1	1	0	1	1	0	1	
*Kalanchoe pinnata* (Lam.) Pers.*	1	1	1	1	1	1	1	0	1	0	0	0	1	1	0	1	1	0	1	0	1	1	1	
*Cucurbita maxima* Duchesne	0	0	0	1	1	0	1	0	0	0	0	0	0	0	0	0	0	0	0	0	0	0	1	Fever, swelling of female genitalia
*Luffa aegyptiaca* Mill	0	0	0	0	0	0	0	0	0	0	0	0	0	0	0	0	0	0	0	0	0	0	1	
*Momordica charantia* L	1	0	0	1	0	0	1	0	1	1	0	1	1	0	1	1	0	0	0	1	1	0	1	
*Cyperus mindorensis* (Steud.) Huygh	0	0	0	0	0	1	0	0	0	0	0	0	0	0	0	0	0	0	0	0	0	0	1	
*Dioscorea esculenta* (Lour.) Burkill	0	0	0	0	1	0	1	0	0	0	0	0	0	0	0	0	0	0	0	0	0	0	1	Mumps, ringworm
*Euphorbia hirta* L.*	1	1	1	0	0	1	1	0	0	1	0	0	0	1	1	1	1	1	1	1	1	0	1	UTI, angular cheilitis
*Euphorbia tirucalli* L.	0	0	0	0	0	0	0	0	0	0	0	0	0	0	0	0	0	0	0	0	0	0	1	Tooth decay
*Jatropha curcas* L.	1	1	1	1	1	1	1	0	1	1	0	0	1	1	1	1	1	0	1	1	1	1	1	Tetanus
*Manihot esculenta* Crantz	0	0	1	1	1	1	1	0	0	0	0	0	0	0	0	0	0	0	1	0	0	0	1	Fracture, gas pain, lower back pain
*Caesalpinia sappan* L.*	0	1	0	0	0	0	1	0	0	1	0	0	0	1	0	0	0	0	0	1	1	1	1	Typhoid fever
*Cajanus cajan* (L.) Huth	0	0	0	0	1	0	1	0	0	0	0	0	1	0	0	0	0	0	0	0	0	0	1	
*Clitoria ternatea* L.	0	0	1	0	0	0	0	0	0	0	0	0	0	0	0	0	0	0	0	0	0	0	1	Cancer, hypertension
*Desmodium triflorum* (L.) DC	0	0	0	0	0	0	0	0	0	0	0	0	0	0	0	1	0	0	0	1	0	0	1	
*Gliricidia sepium (Jacq.)* Kunth ex Walp	0	0	1	1	1	0	1	0	0	0	0	1	1	1	0	1	1	0	0	1	0	0	1	
*Indigofera tinctoria* L	0	0	0	0	0	1	0	0	0	0	0	0	0	0	0	0	0	0	0	1	0	0	1	
*Leucaena leucocephala* (Lam.) de Wit	0	1	0	0	0	0	1	0	0	0	0	0	1	1	0	1	0	1	0	0	0	0	1	
*Mimosa pudica* L.*	0	0	1	1	1	1	1	0	1	1	0	1	1	1	0	0	1	1	1	1	0	1	1	
*Phaseolus lunatus* L.	0	0	0	0	1	0	1	0	0	0	0	0	1	0	0	0	0	0	0	0	0	0	1	
*Pithecellobium dulce* (Roxb.) Benth.	0	0	1	0	1	0	1	0	0	0	0	0	1	0	0	0	0	0	0	1	0	0	1	
*Senna alata* (L.) Roxb.*	1	0	0	1	0	0	1	1	1	0	0	1	1	1	1	0	0	0	1	1	0	0	1	
*Tamarindus indica* L.	0	0	0	0	1	0	1	0	1	0	0	0	1	0	0	0	0	0	1	1	0	0	1	
*Vigna unguiculata* (L.) Walp	0	0	0	0	1	0	0	0	0	0	0	0	0	0	0	0	0	0	0	0	0	0	1	Bloated stomach/gas pain, gas pain
*Cratoxylum sumatranum* (Jack) Blume*	0	0	0	0	0	0	0	0	0	1	0	0	0	0	0	0	0	0	0	1	0	0	1	
*Eleutherine palmifolia* (L.) Merr.	**0**	**0**	**0**	**0**	**0**	**0**	**0**	**0**	**0**	**0**	**0**	**0**	**0**	**0**	**0**	**0**	**0**	**0**	**0**	**0**	**0**	**0**	**0**	Rashes, headache
*Clerodendrum quadriloculare* (Blanco) Merr.	0	0	0	0	0	0	1	0	0	0	0	0	0	0	0	1	0	0	0	1	0	0	1	
*Gmelina arborea* Roxb. ex Sm*.*	0	0	0	0	1	1	0	0	0	1	0	0	0	1	0	1	1	0	0	1	1	0	1	
*Hyptis capitata* Jacq.	0	0	0	0	1	0	0	0	0	0	0	0	1	0	0	1	1	0	0	1	0	0	1	Helminthiasis
*Mentha arvensis* L.	1	0	1	1	0	1	0	0	0	0	0	0	0	0	1	0	1	0	1	0	1	0	1	
*Orthosiphon aristatus* (Blume) Miq.	0	0	0	1	1	0	0	0	0	0	0	0	0	0	0	0	1	0	0	0	0	0	1	Cancer
*Plectranthus amboinicus* (Lour.) Spreng.	0	1	0	0	1	0	1	0	0	0	0	1	0	0	0	1	1	0	1	1	1	0	1	
*Plectranthus scutellarioides* (L.) R.Br.	0	1	0	1	0	0	1	0	1	0	0	0	1	1	0	1	1	0	1	1	0	1	1	
*Tectona grandis* L.f.	0	0	0	0	0	0	0	0	0	0	0	0	1	0	0	0	0	0	0	0	0	0	1	Cuts, wounds, rheumatoid arthritis
*Vitex trifolia* L.*	0	1	0	0	0	0	1	0	0	0	0	0	0	0	0	1	0	0	0	1	0	0	1	
*Persea americana* Mill	1	1	0	1	1	1	1	0	1	1	0	1	1	1	0	1	0	0	1	0	1	1	1	
*Barringtonia asiatica* (L.) Kurz	0	0	0	1	0	0	1	0	0	0	0	0	0	0	0	1	0	0	0	0	0	0	0	
*Lygodium circinnatum* (Burm. f.) Sw.*	0	0	0	0	0	0	0	0	0	0	0	0	0	0	0	0	0	0	0	0	0	0	1	Headache
*Lagerstroemia speciosa* (L.) Pers.	1	0	0	1	1	0	1	0	1	0	0	1	1	1	0	0	1	0	1	1	1	0	1	Cancer
*Abelmoschus esculentus* (L.) Moench	0	0	1	0	0	0	0	0	0	0	0	0	0	0	0	0	0	0	0	0	0	0	1	UTI, cancer, hypertension
*Corchorus olitorius* L.	0	0	1	0	0	0	1	0	0	0	0	0	0	0	0	0	0	0	0	0	0	0	1	Birth control
*Hibiscus acetosella* Welw. ex Hiern*	**0**	**0**	**0**	**0**	**0**	**0**	**0**	**0**	**0**	**0**	**0**	**0**	**0**	**0**	**0**	**0**	**0**	**0**	**0**	**0**	**0**	**0**	**0**	Cuts, wounds, boils, anemia, hypertension, diabetes, rhinorrhea, cough
*Urena lobata* L.*	0	0	0	0	1	0	1	0	0	1	0	0	0	0	0	1	1	0	1	0	0	0	1	
*Sandoricum koetjape* (Burm.f.) Merr.	0	0	1	0	1	0	1	0	1	1	0	0	1	1	1	1	1	0	1	0	1	0	1	Kidney trouble
*Swietenia mahagoni* (L.) Jacq	0	1	1	0	1	0	1	0	0	0	0	1	1	0	1	1	1	0	0	0	0	0	1	
*Tinospora crispa* (L.) Hook. f. and Thomson*	0	1	0	0	0	0	1	0	0	0	0	1	0	0	1	1	1	0	0	1	1	0	1	
*Artocarpus heterophyllus* Lam	0	1	1	0	1	1	1	0	0	0	0	1	1	1	1	0	0	0	0	0	0	0	1	
*Ficus benjamina* L*.* HNUL 0021383	0	1	0	0	0	1	0	0	0	1	0	0	0	1	0	1	0	0	1	1	0	0	1	Postpartum care and recovery, stomachache
*Ficus septica* Burm.f.	0	0	0	1	0	0	1	1	0	0	0	1	0	1	0	1	1	0	0	1	1	0	1	
*Moringa oleifera* Lam.	0	1	1	1	1	1	1	0	1	0	0	1	1	0	0	1	0	0	1	1	1	1	1	
*Muntingia calabura* L.	0	0	0	0	1	1	0	0	0	0	0	0	1	1	0	0	1	0	0	0	1	0	1	
*Musa balbisiana* cv. Colla	0	0	0	0	1	0	1	0	0	0	0	1	0	0	0	0	0	0	0	0	0	0	1	
*Musa textilis* Née	0	1	0	0	0	0	0	0	0	1	0	0	0	0	0	0	0	0	0	0	0	0	1	
*Musa* x *paradisiaca* L.	0	1	0	1	1	1	1	0	1	1	0	0	1	0	1	1	0	0	0	1	1	0	1	
*Psidium guajava* L.	1	1	1	1	1	1	1	1	1	1	0	1	1	1	1	1	1	0	1	1	1	1	1	
*Syzygium cumini* (L.) Skeels	0	0	1	0	1	0	1	0	1	1	0	0	1	1	0	1	0	0	0	1	0	0	1	
*Averrhoa bilimbi* L.	0	0	1	1	1	0	1	0	1	0	0	1	1	0	1	1	0	0	1	0	0	0	1	Kidney trouble
*Averrhoa carambola* L.	0	0	1	0	0	1	0	0	0	0	0	0	0	0	0	0	0	0	0	0	0	0	1	Kidney trouble, postpartum care and recovery
*Oxalis triangularis* A.St.-Hil	**0**	**0**	**0**	**0**	**0**	**0**	**0**	**0**	**0**	**0**	**0**	**0**	**0**	**0**	**0**	**0**	**0**	**0**	**0**	**0**	**0**	**0**	**0**	Cuts/wounds
*Peperomia pellucida* (L.) Kunth	0	0	1	0	0	0	0	0	1	0	0	0	0	1	0	0	0	1	1	0	1	1	1	
*Piper betle* L	1	0	1	1	0	0	1	0	1	1	0	0	0	0	0	1	0	0	1	1	0	0	1	Mumps
*Antidesma bunius* (L.) Spreng	0	0	1	1	1	0	1	0	1	0	0	0	1	0	1	0	0	0	0	1	0	0	1	
*Bambusa spinosa* Roxb	0	0	0	0	0	0	0	0	0	0	0	1	1	0	0	1	0	0	0	0	0	0	1	Cancer
*Chrysopogon aciculatus* (Retz.) Trin	0	0	0	0	0	0	1	0	0	0	0	0	0	0	0	0	0	0	0	0	0	0	1	Stomachache, tooth decay, wart
*Cymbopogon citratus* (DC.) Stapf	1	1	0	0	0	1	0	0	1	1	0	1	1	1	0	1	0	0	0	1	1	1	1	Halitosis
*Eleusine indica* (L.) Gaertn.*	0	1	0	0	1	1	1	0	1	1	0	0	1	1	0	0	1	1	0	1	1	1	1	
*Imperata cylindrica* (L.) Raeusch	1	1	0	1	1	1	1	0	1	1	0	0	1	1	1	1	1	1	1	1	0	1	1	Cancer
*Oryza sativa* L	0	0	0	1	1	0	0	0	0	0	0	0	0	0	0	1	0	0	0	1	0	1	1	Postpartum care and recovery
*Saccharum officinarum* L.	0	0	1	1	1	0	0	0	1	0	0	0	0	0	0	1	0	0	0	0	0	0	1	Hoarseness
*Zea mays* L.	1	1	0	1	1	1	1	0	0	0	0	0	0	0	0	0	0	0	0	0	1	1	1	
*Chrysophyllum cainito* L.	0	1	1	1	0	1	1	0	1	1	0	1	1	1	1	1	0	0	0	1	0	1	1	
*Capsicum annuum* L.	1	1	1	1	1	1	1	0	1	0	0	0	0	1	1	1	1	0	1	0	1	0	1	
*Solanum lycopersicum* L.	1	0	0	1	1	0	0	0	0	0	0	0	0	0	0	0	0	0	0	1	0	0	1	
*Nicotiana tabacum* L.	0	1	0	1	1	0	0	0	0	0	0	0	0	0	0	0	0	0	0	1	0	0	1	
*Solanum melongena* L.	0	0	0	1	1	1	1	0	0	0	0	0	1	0	0	0	0	0	0	1	0	0	1	
*Nauclea orientalis* (L.) L.	0	0	1	0	0	0	1	0	0	0	0	0	0	0	0	1	0	0	0	1	0	0	1	
*Morinda citrifolia* L.	0	0	1	1	1	0	1	1	0	1	0	0	0	0	0	1	0	0	0	1	0	0	1	
*Mussaenda philippica* A.Rich	1	0	1	0	0	0	0	0	0	0	0	0	1	0	0	1	1	0	0	0	0	0	1	
*Citrus maxima* (Burm.) Merr.	0	0	0	0	1	0	1	0	0	0	0	1	1	1	1	1	0	0	0	1	1	0	1	Kidney trouble, chicken pox
*Citrus microcarpa* Bunge	1	1	1	0	0	1	1	0	1	0	0	1	0	1	1	1	0	0	0	1	1	0	1	
*Pipturus arborescens* (Link) C.B. Rob.*	0	0	0	0	1	0	0	0	0	0	0	0	0	0	0	0	1	0	0	0	1	0	1	Breast engorgement
*Stachytarpheta jamaicensis* (L.)*	0	1	0	1	1	1	0	1	0	1	0	0	0	0	1	1	1	1	1	1	1	0	1	
*Cissus* sp.																								
*Leea guineensis* G. Don	0	0	0	0	1	0	0	0	0	0	0	0	0	0	0	0	0	0	0	1	1	0	1	
*Tetrastigma* sp. Planch.																								
*Alpinia galanga* (L.) Willd	0	0	0	0	0	0	0	0	0	0	0	0	0	0	0	1	0	0	0	0	0	0	1	
*Amomum* sp.																								
*Curcuma longa* L.	0	1	0	1	0	1	1	0	0	1	0	0	0	1	0	0	1	0	1	0	1	1	1	Typhus, memory loss, tetanus
*Etlingera philippinensis* (Ridl.) R.M.Sm.	0	0	0	0	0	0	0	0	0	0	0	0	0	0	0	0	0	0	0	**1**	0	0	0	Typhoid fever, tuberculosis, headache
*Kaempferia galanga* L.	0	0	0	0	0	0	1	0	0	1	0	0	0	0	0	0	1	0	1	0	1	1	1	
*Zingiber officinale* Roscoe	1	1	0	1	1	1	1	0	1	0	0	1	1	1	1	1	0	0	0	1	1	1	1	Tetanus

1-Balangcod and Balangcod, 2011.

2-Olowa et al., 2012.

3-Tantiado, 2012.

4-Abe and Ohtani, 2013.

5-Ragragio et al., 2013.

6-Gruyal et al., 2014.

7-Ong and Kim, 2014.

8-Raterta et al., 2014.

9-Balangcod and Balangcod, 2015.

10-Pizon et al., 2016.

11-Odchimar et al., 2017.

12-Baddu and Ouano, 2018.

13-Tantengco et al., 2018.

14-Agapin, 2019.

15-Pablo, 2019.

16-Cordero et al., 2020.

17-Dapar et al., 2020.

18-Paraguison et al., 2020.

19-Belgica et al., 2021.

20-Cordero and Alejandro, 2021.

21-Nuñeza et al., 2021.

22-Montero and Geducos, 2021.

23-PTKD (Philippine Traditional Knowledge Digital Library on Health).

*Medicinal plants recorded in *Panay Bukidnon* communities in the Province of Capiz (Jocano, 1968).

Plants in bold are the newly recorded species with medicinal values.

Values in bold indicate the absence of medicinal value of the newly recorded species compared with the other existing ethnobotanical studies in the Philippines.

## Discussion

The documentation of 131 medicinal plant species used in the indigenous health care practices showed the extensive usage of *Panay Bukidnon* ethnobotanical knowledge and indicative importance for their rich cultural heritage. The families of Fabaceae, Lamiaceae, and Poaceae were represented with a high number of medicinal plant species. Fabaceae as the most preferred medicinal plant family used by the *Panay Bukidnon* is parallel to the other folkloric studies conducted in Western Visayas (Madulid et al., 1989; Tantiado 2012; Ong and Kim, 2014; Cordero and Alejandro, 2021) and other indigenous communities in the country (Ragragio et al., 2013; Obico and Ragrario, 2014; Tangtengco et al., 2018; Pablo, 2019). Fabaceae is highly used by the *Panay Bukidnon* to treat infectious and parasitic diseases and diseases and symptoms or signs involving the digestive system or abdomen. The family constitutes phytochemicals that have antibacterial, antifungal, antioxidant, and insecticidal activities (Wanda et al., 2015).

The use of leaves as the most preferred medicinal plant part to address medical conditions is comparable to other ethnobotanical surveys conducted throughout the archipelago (Balangcod and Balangcod, 2011; Olowa et al., 2012; Abe and Ohtani, 2013; Gruyal et al., 2014; Ong and Kim, 2014; Raterta et al., 2014; Balangcod and Balangcod, 2015; Pizon et al., 2016; Odchimar et al., 2017; Baddu and Ouano, 2018; Tantengco et al., 2018; Agapin, 2019; Pablo, 2019; Cordero et al., 2020; Dapar et al., 2020; Belgica et al., 2021; Cordero and Alejandro, 2021; Madjos and Ramos, 2021; Montero and Geducos, 2021; Nuñeza et al., 2021). As a tropical country, leaves are always available for most plant species at all seasons and are readily accessible in case of emergencies. The collection of leaves is more sustainable than gathering other plant parts such as barks and roots that can cause damaging effects and even mortality to a plant if harvested in large quantities. Leaves contain the highest secondary metabolites with an antimicrobial effect (Chanda and Kaneria, 2011), antioxidant property, antibiotic activity, and antidiabetic potential compared with other plant parts (Jain et al., 2019).

Decoction is the most common form of preparation and preferably to be taken orally and occasionally used for body steaming, bathing, and washing. It is also an evident form of preparation in other indigenous communities in the country (Balangcod and Balangcod, 2015; Pizon et al., 2016; Odchimar et al., 2017; Baddu and Ouano, 2018; Tantengco et al., 2018; Cordero et al., 2020; Cordero and Alejandro, 2021; Madjos and Ramos, 2021; Nuñeza et al., 2021). Decoction is done with the use of one medicinal plant species or in a combination of two or more. The *Panay Bukidnon* are culturally used to combine three, five, or seven (colloquially known as *pito-pito*) different medicinal plants for higher efficacy. Each plant constitutes phytochemical compounds and is sometimes present in small quantities and inadequate to achieve desirable therapeutic effects. To yield better results and effectiveness, the combination of different medicinal plants demonstrates the synergistic effects. Some bioactive chemicals work significantly when combined with other plants rather than used singly (Parasuraman et al., 2014).

*Curcuma longa* recorded the highest use value and is used as therapy for headache, fever, body pain, stomachache, bloated stomach, tooth decay, typhus, typhoid fever, anti-tetanus, memory loss, cancer, and *sinda*. The rhizome’s extract is usually used for the treatment. It is also used by other indigenous groups in the country for diarrhea, abdominal pain, flatulence, arthritis, and hypertension by the *Higaonon* tribe in Iligan City (Olowa et al., 2012); arthritis, cough, and cuts and wounds by the *Ivatan* tribe in Batan Island (Abe and Ohtani, 2013); fever, burn, dizziness, and abdominal pain by the *Ati* tribe in Guimaras Island (Ong and Kim, 2014); arthritis by the *Subanen* tribe in Zamboanga del Sur (Pizon et al., 2016); flatulence, headache, numbness, rheumatism, stomachache, and vomiting by the *Aetas* tribe in Bataan (Pablo, 2019); cancer by the *Manobo* tribe in Bukidnon (Pucot et al., 2019); skin eruptions and gastric pain by the *Ati* tribe in Aklan (Cordero et al., 2020); ten different diseases by the *Manobo* tribe in Agusan del Sur (Dapar et al., 2020); myoma, hepatitis, relapse, sore eyes, and stye by the eight ethnolinguistic groups in Zamboanga Peninsula (Madjos and Ramos, 2021); bruise and boils by the *Mamanwa* tribe in Surigao del Norte and Agusan del Norte (Nuñeza et al., 2021). In India, the use of *C. longa* dates back to 4,000 years ago not only as a culinary spice but also for religious and medicinal importance. It contains bioactive compounds that have antioxidant, antimutagenic, antimicrobial, antimutagenic, antimicrobial, antifungal, anticancer, and other countless medicinal uses (Prasad and Aggarwal, 2011).

*Artemisia vulgaris* has the highest relative frequency citation. It is a cosmopolitan weed and is available nearly everywhere. It thus does not surprise that it is commonly used for cough, fever, headache, body pains, chest pain, fracture, and hearing problems. Other ethnobotanical surveys mentioned its efficacy against cough and scabies by the *Kalanguya* tribe in Ifugao (Balangcod and Balangcod, 2011); stomachache (Olowa et al., 2012); sore eyes, ear infection, and cough by the *Ayta* tribes in Pampanga (Ragragio et al., 2013); cough with phlegm, fever, abdominal pain, body pains, and headache (Ong and Kim, 2014); colds by the *Talaandig* tribe in Bukidnon (Odchimar et al., 2017); dysmenorrhea by the *Y’Apayaos* in Cagayan arthritis (Baddu and Ouano, 2018); fever, sore throat, colds, cough, and phlegm *Ayta* in Bataan (Tantengco et al., 2018); fever, headache, dizziness, stomachache, bloated stomach, and cough (Cordero et al., 2020); 11 different folkloric uses (Madjos and Ramos, 2021); cough and gas pain (Cordero and Alejandro, 2021); fever, cough, and cough with phlegm (Nuñeza et al., 2021). In medieval times, it was known as the “mother of herbs” due to its beneficial effects. Studies have been conducted worldwide for its antioxidant, bronchodilatory, hepatoprotective analgesic, antihypertensive, estrogenic, cytotoxic, antifungal and antibacterial, anti-inflammatory, anti-allergenic, antimalarial, and anthelmintic activities (Ekiert et al., 2020). *Artemisia,* per se, is an extremely important plant genus, pharmacologically as well as economically. *A. annua* L. makes the most important example, famous for its many pharmacologically active substances but especially for Artemisin (Tu, 2011), an effective remedy against malaria.

*Annona muricata*, an important, widely grown fruit tree, has the highest relative importance index value (0.88) and is used to treat 13 diseases in 11 different use or disease categories. It recorded the high use report for treating UTI, cancer, and hypertension by drinking the leaf decoction or eating just the ripe fruit. It is also used by the *Panay Bukidnon* for high uric level, pneumonia, dizziness, intestinal cleansing, kidney trouble, *doklong*, itchy throat, amoebiasis, lump, and arthritis. In traditional medicine across the country, it is also used for the treatment of diarrhea (Olowa et al., 2012); dermatological diseases (Tantiado, 2012); fever, insect repellent, headache, and stomachache (Ragragio et al., 2013); tetanus (Pizon et al., 2016); gastrointestinal cleansing and tumors (Odchimar et al., 2017); fever and arthritis (Baddu and Ouano, 2018); stomachache and dizziness (Tantengco et al., 2018); diabetes, high blood, stomachache, UTI, and vertigo (Pablo, 2019); cancer (Agapin, 2019); 12 different diseases (Dapar et al., 2020); kidney problems, urinary tract infection, goiter, and anthelmintic (Cordero et al., 2020); at least 16 medical problems (Madjos and Ramos, 2021); cuts and wounds, stomach ulcer, intestinal cleansing, UTI, cough, and cancer (Cordero et al., 2020); cancer (Montero and Geducos, 2021); cough, wound, asthma, and UTI (Nuñeza et al., 2021); cancer, stomach acidity, hypertension, and cough (Belgica et al., 2021). Phytochemical constituents investigated on *A. muricata* exhibited antiarthritic, anticancer, anticonvulsant, antidiabetic, anti-inflammatory, antioxidant, antihypertensive, antiparasitic, antiplasmodial, cytotoxic, gastroprotective, and wound healing activity (Moghadamtousi et al., 2015; Coria-Téllez et al., 2018).

The highest ICF value is in the diseases of the respiratory system category and *Blumea balsamifera* is the most frequently used medicinal plant to treat cough. A high number of informants agreed on the effectiveness of *B. balsamifera* against the diseases on the respiratory system, particularly for treating cough in the community. However, this therapeutic claim must be seriously considered for further pharmacological investigations to determine its efficacy. *B. balsamifera* is one of the ten medicinal plants endorsed by the Philippine Department of Health (DOH) as part of basic healthcare and clinically proven to have diuretic and antiurolithiasis properties. It is manufactured in the country for national distribution and marketing by the National Drug Formulary (World Health Organization, 1998). It also contains compounds (monoterpenes, diterpenes, sesquiterpenes) that have antitumor, antioxidant, antimicrobial and anti-inflammation, antiplasmodial, antityrosinase, wound healing, anti-obesity, disease and insect resistance, and hepatoprotective effects and radical scavenging activities (Pang et al., 2014).

The medicinal plant with the highest FL was the *Chromolaena odorata* under the injury, poisoning, and certain other consequences of external causes category. All informants who cited *C. odorata* preferred to use it as first aid for cuts and wounds. This suggests that *C. odorata* might contain valuable bioactive compounds with pharmacological effects for cuts and wounds that must be proven scientifically. Several ethnobotanical studies also recorded the use of *C. odorata* for cuts and wounds in the country (Olowa et al., 2012; Ong and Kim, 2014; Pizon et al., 2016; Odchimar et al., 2017; Tantengco et al., 2018; Cordero et al., 2020; Dapar et al., 2020; Belgica et al., 2021; Cordero and Alejandro 2021; Madjos and Ramos, 2021). The leaves of *C. odorata* are rich in flavonoids and have the highest concentration of allelochemicals. They have antimalarial, anti-inflammatory, antibacterial, analgesic, antipyretic, antioxidant, anticancer, and wound healing properties (Vijayaraghavan et al., 2017).

Traditional medical practices in the indigenous groups in the Philippines are generally influenced by their cultural, spiritual, and religious beliefs of supernatural beings. *Curcuma longa* is the most preferred medicinal plant administered by the *Panay Bukidnon* to a sick person with conditions caused by unseen beings. In Hindu worship rights, *C. longa* has been used for offerings and magic (Velayudhan et al., 2012).

Plant-based compounds have been in constant use since ancient times for any emerging disease. There were several bioactive compounds extracted from medicinal plants with promising antiviral properties against the novel coronavirus (COVID-19) (Adhikari et al., 2020). In Thailand, 60 medicinal plant species were used to treat mild symptoms of COVID-19 (Phumthum et al., 2021). In Nepal, there were also 60 medicinal plants used (Khadka et al., 2021) and 23 plants in Morroco (El Alami et al., 2020) for potential COVID-19 therapy and *Zingiber officinale* is one of the common species used. In Bangladesh, phytochemicals extracted from *Calotropis gigantea* exhibited positive inhibitory effects against the COVID-19 virus (Dutta et al., 2021), as well as the alkaloids and terpenoids isolated from plants of African origin (Gyebi et al., 2021). *Curcumin* from *C. longa* also showed promising effects against the virus (Adhikari et al., 2020).

In the Philippines, the Department of Science and Technology (DOST) has been conducted clinical trials and explored the therapeutic effects of the virgin coconut oil (VCO), *Euphorbia hirta* (tawa-tawa), and *Vitex trifolia* (lagundi) for their potential efficacy against COVID-19 infection (Arayata, 2021). In the recent updates published in the Global Media Arts (GMA) news articles, clinical trials for *V. trifolia* and *E. hirta* have been proven to decrease mild-to-moderate symptoms of COVID-19. Mild-to-moderate symptoms of 172 random COVID-19 patients disappeared within 3–5 days after taking a 1,950 mg capsule of *E. hirta* thrice a day for ten days as a food supplement. *V. trifolia* also showed a positive result in decreasing mild symptoms of COVID-19. Community trials of VCO as an adjuvant for mild symptoms of COVID-19 patients showed a positive result in decreasing the virus count by 60–90%. A clinical trial of VCO on mild and severe symptoms of COVID-19 conducted in Philippine General Hospital is still ongoing (Global Media Arts News, 2021a; Global Media Arts News, 2021b).

*V. trifolia*, *C. longa, Z. officinale, Capsicum annuum, E. hirta,* and *Peperomia pellucida* were used by the *Panay Bukidnon* as an alternative medicine to strengthen their immunity and they have claimed that these species can alleviate the symptoms of the COVID-19 infection. They used these plants traditionally to treat fever, headache, cough, and body pains, which were also the common indications of COVID-19. Further pharmacological research and investigations are highly suggested for these medicinal plants to explore their potential uses and therapeutic effects against COVID-19 infection especially for *Zingiber officinale, Capsicum annuum*, and *Peperomia pellucida* species*.* The *Panay Bukidnon* also believed that chewing betel quid could give them the strength to fight the virus. Chewing betel quid has been a customary practice of Filipinos since the pre-Spanish colonial period throughout the Philippines. It is part of the social undertakings and ceremonies and is believed to increase stamina, good health, and longevity (Valdes, 2004). In India, the practice of chewing betel dates back to around 75 AD and it is known for centuries for its therapeutic properties (Toprani and Patel 2013). A review was conducted on the synergistic prophylaxis effects of *Piper betle* and gold ash can hypothetically limit and manage the COVID-19 infection (Sharma and Malik, 2020).

For the comparative review performed on the medicinal plants with other ethnobotanical studies conducted in the country, three species (*Eleutherine palmifolia*, *Hibiscus acetosella*, and *Oxalis triangularis*) showed no record of medicinal value in the previous studies. *E. palmifolia* is used by the *Dayaks* in Indonesia to treat a variety of diseases such as diabetes, cancer, hypertension, stroke, and sexual disorders and as a galactagogue. Bioactive compounds from this species contain various pharmacological activities such as antibacterial, anti-inflammatory, anticancer, and antidiabetic (Kamarudin et al., 2021). *H. acetosella* is used as therapy for anemia in Southern Uganda (Ssegawa and Kasenene, 2007) and its phenolic compounds exhibit antioxidant and antibacterial properties (Lyu et al., 2020). Limited literature is available for *O. triangularis,* but its medicinal uses include remedies for fever, UTI, mouth sores, cuts, rashes, and skin infections (Arakelyan and Arakelyan, 2020). The comparison of the medicinal plants and their uses was performed with five ethnobotanical studies that were previously conducted in the rural and urban communities and villages (Tantiado, 2012; Gruyal et al., 2014; Agapin, 2019; Belgica et al., 2021; Montero and Geducos, 2021), 17 studies conducted in the IP communities (Balangcod and Balangcod, 2011; Olowa et al., 2012; Abe and Ohtani, 2013; Ragragio et al., 2013; Ong and Kim, 2014; Raterta et al., 2014; Balangcod and Balangcod, 2015; Pizon et al., 2016; Odchimar et al., 2017; Baddu and Ouano, 2018; Tantengco et al., 2018; Pablo, 2019; Cordero et al., 2020; Dapar et al., 2020; Paraguison et al., 2020; Cordero and Alejandro, 2021; Nuñeza et al., 2021) all over the country, and one online database: the Philippine Traditional Knowledge Digital Library on Health (Philippine Traditional Knowledge Digital Library on Health, 2021). The PTKDL is an electronic library that documented 16,690 enumerations of medicinal plant preparations and 66 healing rituals and practices mentioned by 509 traditional healers in 43 different research sites in the country (World Health Organization, 2019). The database (https://www.tkdlph.com/) recorded about 1,200 medicinal plants used by the local and indigenous communities from different ethnobotanical studies, lexicographic and linguistic texts, and current researches conducted in selected indigenous communities nationwide.

## Conclusion

The ethnobotanical use of many different plant species is an important predominating practice in the Philippines. It is an integral part of Filipino custom and tradition and has been culturally accepted for ages. The results of this ethnobotanical documentation of 131 medicinal plants used in addressing 91 diseases across 16 different disease categories portray the strong dependence of the *Panay Bukidnon* in the medicinal flora in their area. This could be attributed to the great distance of the study site to the town and the health centers or well-functioning hospitals. The most culturally relevant and important species recorded in this study in terms of UV, RFC, RI, ICF, and FL are *Curcuma longa*, *Blumea balsamifera*, *Artemisia vulgaris, Annona muricata,* and *Chromolaena odorata,* respectively. The efficacy and effectivity of the therapeutic claims of these species must be further pharmacologically investigated and validated. These species have been used for centuries by many people worldwide and have proven to cure a myriad of diseases. The comparative study of the medicinal plants with other ethnobotanical studies revealed some novel and additional therapeutic uses that are valuable to the immense body of traditional knowledge and practices in the country. The traditional knowledge and practices from indigenous peoples add more treatment opportunities for potential therapy of pre-existing and novel diseases. The indigenous knowledge on the medicinal plants used by the *Panay Bukidnon* is passed from one generation to the other mostly in oral forms with the influence of their religious and cultural beliefs. Furthermore, it is urgent to document the indigenous knowledge before it is forgotten because of environmental and social challenges such as species extinction, climate change, acculturation, modernization, availability and accessibility of prescribed medicines, and lack of interest of the younger generations. The results of this study also serve as a medium for preserving cultural heritage, ethnopharmacological bases for further drug research and discovery, and preserving biological diversity. The ethnobotanical study on the *Panay Bukidnon* communities in Panay Island is limited by the expensive and lengthy process of acquiring government permits and by the fact that some communities are infested by leftists (New People’s Army) that could risk the safety of researchers and there are no access roads in the upland areas. Lastly, it is strongly recommended to conduct further comprehensive surveys on other *Panay Bukidnon* communities in other provinces of the Panay Island and to conduct pharmacological studies and investigations on the important medicinal plants, especially the ones that have high ICF and FL values for potential drug development and formulation.

## Data Availability

The original contributions presented in the study are included in the article/Supplementary Material, further inquiries can be directed to the corresponding author.
