# Supplementary material for: Ethnobotanical Documentation of Medicinal Plants Used by the Indigenous Panay Bukidnon in Lambunao, Iloilo, Philippines
Source: Front Pharmacol. 2022 Jan 10;12:790567. doi: 10.3389/fphar.2021.790567 (PMC8784692; doi:10.3389/fphar.2021.790567)
Supplement: Supplementary file 1 [file Table1.pdf]

Supplementary Table

Table 1. Medicinal plants growth form, collection sites, and geographical distribution and endemicity.

**Scientific Name**	**Growth Form**	**Collection Site**	**Geographical Distribution and Endemicity**
Andrographis paniculata (Burm.f.) Nees	Herb	Cultivated	Not Native
Justicia gendarussa Burm.f.	Shrub	Cultivated	Native
Pseuderanthemum carruthersii (Seem.) Guillaumin	Shrub	Cultivated	Not Native
Acorus calamus L.	Herb	Cultivated	Not Native
Amaranthus viridis L.	Herb	Cultivated	Not Native
Alternanthera sessilis (L.) R. Br. ex DC.	Herb	Cultivated	Native
Allium sativum L.	Herb	Cultivated	Not Native
Allium fistulosum L.	Herb	Cultivated	Not Native
Mangifera indica L.	Tree	Cultivated	Not Native
Spondias pinnata (L.f.) Kurz	Tree	Cultivated	Native
Annona muricata L.	Tree	Cultivated	Not Native
Annona squamosa L.	Tree	Cultivated	Not Native
Centella asiatica (L.) Urb.	Herb	Wild	Native
Alstonia scholaris (L.) R.Br.	Tree	Wild	Native
Catharanthus roseus (L.) G.Don	Shrub	Cultivated	Not Native
Tabernaemontana pandacaqui Poir.	Shrub	Wild	Native
Alocasia macrorrhizos (L.) G.Don	Herb	Wild	Native
Alocasia cultivar	Herb	Cultivated	-
Colocasia esculenta (L.) Schott	Herb	Cultivated	Native
Homalomena philippinensis Engl.	Herb	Wild	Native
Schefflera elliptica (Blume) Harms	Shrub	Cultivated	Native
Areca catechu L.	Tree	Cultivated	Native - Endemic
Cocos nucifera L.	Tree	Cultivated	Native
Corypha utan Lam.	Tree	Wild	Native
Cordyline fruticosa (L.) A.Chev.	Shrub	Cultivated	Not Native
Aloe vera (L.) Burm.f.	Herb	Cultivated	Not Native
Artemisia vulgaris L.	Herb	Cultivated	Not Native
Bidens pilosa L.	Herb	Wild	Not Native
Blumea balsamifera (L.) DC.	Shrub	Cultivated	Native
Chromolaena odorata (L.) R.M.King & H.Rob.	Shrub	Wild	Not Native
Elephantopus tomentosus L.	Herb	Wild	Not Native
Impatiens balsamina L.	Herb	Cultivated	Not Native
Basella alba L.	Herb	Cultivated	Native
Bixa orellana L.	Tree	Cultivated	Not Native
Cordia dichotoma G.Forst.	Tree	Wild	Native
Brassica rapa L.	Herb	Cultivated	Not Native

Ananas comosus (L.) Merr.	Herb	Cultivated	Not Native
Carica papaya L.	Tree	Cultivated	Not Native
Ipomoea batatas (L.) Lam.	Herb	Cultivated	Not Native
Decalobanthus peltatus (L.) A.R.Simões & Staples	Climber	Wild	Native
Cheilocostus speciosus (J.Koenig) C.D.Specht	Herb	Wild	Native
Kalanchoe pinnata (Lam.) Pers.	Herb	Cultivated	Not Native
Cucurbita maxima Duchesne	Climber	Cultivated	Not Native
Luffa aegyptiaca Mill.	Climber	Cultivated	Not Native
Momordica charantia L.	Climber	Cultivated	Native
Cyperus mindorensis (Steud.) Huygh	Herb	Wild	Native
Dioscorea esculenta (Lour.) Burkill	Climber	Cultivated	Native
Euphorbia hirta L.	Herb	Wild	Not Native
Euphorbia tirucalli L.	Shrub	Cultivated	Not Native
Jatropha curcas L.	Tree	Cultivated	Not Native
Manihot esculenta Crantz	Shrub	Cultivated	Not Native
Caesalpinia sappan L.	Tree	Wild	Not Native
Cajanus cajan (L.) Huth	Shrub	Cultivated	Not Native
Clitoria ternatea L.	Climber	Cultivated	Not Native
Desmodium triflorum (L.) DC	Herb	Wild	Native
Gliricidia sepium (Jacq.) Kunth ex Walp	Tree	Cultivated	Not Native
Indigofera tinctoria L.	Shrub	Wild	Not Native
Leucaena leucocephala (Lam.) de Wit	Tree	Wild	Not Native
Mimosa pudica L.	Herb	Wild	Not Native
Phaseolus lunatus L.	Climber	Cultivated	Not Native
Pithecellobium dulce (Roxb.) Benth.	Tree	Cultivated	Not Native
Senna alata (L.) Roxb.	Shrub	Wild	Not Native
Tamarindus indica L.	Tree	Cultivated	Not Native
Vigna unguiculata (L.) Walp.	Climber	Cultivated	Not Native
Cratoxylum sumatranum (Jack) Blume	Tree	Cultivated	Native
Eleutherine palmifolia (L.) Merr.	Herb	Cultivated	Not Native
Clerodendrum quadriloculare (Blanco) Merr.	Shrub	Cultivated	Native
Gmelina arborea Roxb. ex Sm.	Tree	Wild	Not Native
Hyptis capitata Jacq.	Herb	Wild	Not Native
Mentha arvensis L.	Herb	Cultivated	Not Native
Orthosiphon aristatus (Blume) Miq.	Shrub	Cultivated	Native
Plectranthus amboinicus (Lour.) Spreng.	Herb	Cultivated	Not Native
Plectranthus scutellarioides (L.) R.Br.	Herb	Cultivated	Native
Tectona grandis L.f.	Tree	Wild	Not Native
Vitex trifolia L.	Shrub	Cultivated	Native
Persea americana Mill.	Tree	Cultivated	Not Native
Barringtonia asiatica (L.) Kurz	Tree	Cultivated	Native

Lygodium circinnatum (Burm. f.) Sw.	Climber	Wild	Native
Lagerstroemia speciosa (L.) Pers.	Tree	Wild	Native
Abelmoschus esculentus (L.) Moench	Herb	Cultivated	Not Native
Corchorus olitorius L.	Herb	Cultivated	Native
Hibiscus acetosella Welw. ex Hiern	Herb	Cultivated	Not Native
Urena lobata L.	Herb	Wild	Native
Sandoricum koetjape (Burm.f.) Merr.	Tree	Cultivated	Native
Swietenia mahagoni (L.) Jacq.	Tree	Cultivated	Not Native
Tinospora crispa (L.) Hook. f. & Thomson	Climber	Cultivated	Native
Artocarpus heterophyllus Lam.	Tree	Cultivated	Not Native
Ficus benjamina L. HNUL 0021383	Tree	Cultivated	Native
Ficus septica Burm.f.	Tree	Wild	Native
Moringa oleifera Lam.	Tree	Cultivated	Not Native
Muntingia calabura L.	Tree	Cultivated	Not Native
Musa balbisiana cv. Colla	Herb	Cultivated	Native
Musa textilis Née	Herb	Cultivated	Native - Endemic
Musa x *paradisiaca* L.	Herb	Cultivated	Native
Psidium guajava L.	Tree	Cultivated	Not Native
Syzygium cumini (L.) Skeels	Tree	Cultivated	Not Native
Averrhoa bilimbi L.	Tree	Cultivated	Not Native
Averrhoa carambola L.	Tree	Cultivated	Not Native
Oxalis triangularis A.St.-Hil.	Herb	Cultivated	Not Native
Peperomia pellucida (L.) Kunth	Herb	Wild	Not Native
Piper betle L.	Climber	Cultivated	Native
Antidesma bunius (L.) Spreng	Tree	Wild	Native
Bambusa spinosa Roxb.	Tree	Wild	Not Native
Chrysopogon aciculatus (Retz.) Trin.	Herb	Wild	Native
Cymbopogon citratus (DC.) Stapf	Herb	Cultivated	Not Native
Eleusine indica (L.) Gaertn.	Herb	Wild	Not Native
Imperata cylindrica (L.) Raeusch.	Herb	Wild	Not Native
Oryza sativa L.	Herb	Cultivated	Not Native
Saccharum officinarum L.	Herb	Cultivated	Not Native
Zea mays L.	Herb	Cultivated	Not Native
Chrysophyllum cainito L.	Tree	Cultivated	Not Native
Capsicum annuum L.	Herb	Cultivated	Not Native
Solanum lycopersicum L.	Herb	Cultivated	Not Native
Nicotiana tabacum L.	Herb	Cultivated	Not Native
Solanum melongena L.	Shrub	Cultivated	Not Native
Nauclea orientalis (L.) L.	Tree	Wild	Native
Morinda citrifolia L.	Tree	Cultivated	Native
Mussaenda philippica A.Rich.	Shrub	Wild	Native - Endemic

Citrus maxima (Burm.) Merr.	Tree	Cultivated	Not Native
Citrus microcarpa Bunge	Shrub	Cultivated	Not Native
Pipturus asper Wedd.	Shrub	Wild	Native
Stachytarpheta jamaicensis (L.)	Herb	Wild	Not Native
Cissus sp.	Climber	Cultivated	-
Leea guineensis G. Don	Shrub	Wild	Native
Tetrastigma sp. Planch.	Climber	Cultivated	-
Alpinia galanga (L.) Willd.	Herb	Cultivated	Native
Amomum sp.	Herb	Wild	-
Curcuma longa L.	Herb	Cultivated	Not Native
Etlingera philippinensis (Ridl.) R.M.Sm.	Herb	Wild	Native
Kaempferia galanga L.	Herb	Cultivated	Not Native
Zingiber officinale Roscoe	Herb	Cultivated	Not Native

Note: Medicinal plants identified by the genus level were not assessed for its geographical distribution and endemicity
