# Supplementary material for: Ethnobotanical Documentation of Medicinal Plants Used by the Indigenous Panay Bukidnon in Lambunao, Iloilo, Philippines
Source: Front Pharmacol. 2022 Jan 10;12:790567. doi: 10.3389/fphar.2021.790567 (PMC8784692; doi:10.3389/fphar.2021.790567)
Supplement: Supplementary file 2 [file Table2.docx]

Table 2. Medicinal plants used by the indigenous *Panay Bukidnon* in Brgy. Caguisanan, Lambunao, Iloilo, Philippines.

| **Scientific Name and Accession Number** | **Local Name** | **Family Name** | **UV** | **RFC** | **RI** | **Part Used** | **Disease or Purpose^a^** | **Preparation and administration** | **Quantity** | | **Dosage** | | **Adverse or side effect** |  |
| --- | --- | --- | --- | --- | --- | --- | --- | --- | --- | --- | --- | --- | --- | --- |
| *Andrographis paniculata* (Burm.f.) Nees HNUL 0021421 | Sin-san-soy | Acanthaceae | 0.04 | 0.03 | 0.07 | Leaf | Stomachache, diarrhea, nausea | Drink decoction | Handful of leaves | | Once a day | | Can cause miscarriage in pregnant woman |  |
| *Justicia gendarussa* Burm.f. HNUL 0021359 | Bunlaw | Acanthaceae | 0.52 | 0.37 | 0.6 | Leaf | *Sinda* | Apply on the forehead with *P. carruthersii, Z. officinale*, and *C. longa;* pound with *A. calamus, P. betle, C. longa, V. unguiculata, Z. officinale,* and *A. sativum* then rub extract on the body; apply leaves on the abdomen | 7 leaves | | Once or thrice a day | | None |  |
|  |  |  |  |  |  | Leaf | Headache, fever, dizziness | Apply on the forehead; apply with *P. carruthersii, C. longa, and Z. officinale* or with *B. balsamifera* on the forehead | 5 or 7 leaves | | Once or twice (morning and night) or thrice a day | |  |  |
|  |  |  |  |  |  | Leaf | *Doklong* | Boil and bath decoction | 7 leaves | | Once after being recovered from illness | |  |  |
|  |  |  |  |  |  | Leaf | Postpartum care and recovery | Boil with *B. balsamifera*, *C. sumatranum*, *and B. spinosa* then drink one glass and the rest is for body steaming and bathing; Boil with *I. cylindrica*, *B. spinosa*, *C. maxima*, and *A. bunius* or with *C. citratus*, or *A. carambola*, or *A. squamosa*, or *P. guajava* then drink one glass and bath the rest | 7 leaves | | To be performed nine days after giving birth | |  |  |
|  |  |  |  |  |  | Leaf | Bloated stomach/gas pain | Apply on the stomach with *B. balsamifera*, *Z. officinale*, and *C. longa* | 7 leaves | | Once a day | |  |  |
|  |  |  |  |  |  | Leaf | *Inaswang* | Apply with *C. sappan*, *B. balsamifera*, and *C. maxima* on the stomach after offering some discreet prayer(oration); apply leaves on the stomach | 7 leaves | | Once | |  |  |
| *Pseuderanthemum carruthersii* (Seem.) Guillaumin HNUL 0021324 | Pasaw | Acanthaceae | 0.52 | 0.4 | 0.58 | Leaf | *Sinda* | Apply with *C. longa*, *Z. officinale*, and *J. gendarussa* on the forehead | 7 leaves | | Once or thrice a day | | None |  |
|  |  |  |  |  |  | Leaf | Headache, fever | Apply on the forehead alone or with *C. longa* and *Z. officinale* | 3, 5, or 7 leaves | | Once or twice (morning and night) a day | |  |  |
|  |  |  |  |  |  | Leaf | Dizziness | Apply on the forehead | 5 or 7 leaves | | Twice a day | |  |  |
|  |  |  |  |  |  | Leaf | Postpartum care and recovery | Boil with *J. gendarussa*, *C. maxima*, *A. carambola,* and *B. spinosa* then drink one glass and the rest is for body steaming and bathing | 7 leaves | | To be performed nine days after giving birth | |  |  |
| *Acorus calamus* L. HNUL 0021371 | Labigan | Acoraceae | 0.12 | 0.05 | 0.19 | Rhizome; Leaf | Fever | Pound leaf and rhizome with *C. longa* and rub extract on the body | 7 thin- sliced rhizome | | Once a day | | None |  |
|  |  |  |  |  |  | Rhizome; Leaf | Headache | Pound rhizome or leaf and rub extract on the body | 7 thin-sliced rhizomes | | Once or thrice a day | |  |  |
|  |  |  |  |  |  | Rhizome, leaf | *Sinda* | Pound rhizome or leaf alone or with *P. betle*, *Z. officinale*, *C. longa*, and *A. sativum* then rub extract on the body | 7 thin- sliced rhizome | | Once or thrice a day | |  |  |
| *Amaranthus viridis* L. HNUL 0021317 | Kulitis | Amaranthaceae | 0.03 | 0.03 | 0.07 | Leaf | Anemia | Slightly cook as vegetable and eat | Handful of leaves | | As needed | | None |  |
| *Alternanthera sessilis* (L.) R. Br. ex DC. HNUL 0021384 | Lupo | Amaranthaceae | 0.03 | 0.03 | 0.12 | Leaf | Anemia | Cook as vegetable and eat | Handful of leaves | | As needed | | None |  |
|  |  |  |  |  |  | Leaf | Vomiting blood | Crush and drink extract | 1 spoonful of extract | | Thrice a day | |  |  |
| *Allium sativum* L. HNUL 0021400 | Ahos | Amaryllidaceae | 0.11 | 0.07 | 0.2 | Bulb | Bloated stomach/gas pain, indigestion | Pound with *O. sativa* grains then apply on the stomach; pound heated bulb and apply on the stomach | 3 cloves | | Once a day | | Skin irritation and bad breath |  |
|  |  |  |  |  |  | Bulb | Tooth decay | Crush and insert in decaying tooth | A pinch of clove | | Once a day | |  |  |
|  |  |  |  |  |  | Bulb | Wart | Pound and apply | 1 clove | | Thrice a day for 7  days | |  |  |
|  |  |  |  |  |  | Bulb | *Kolebra* | Chew or pound then eat; pound and rub extract on the stomach | 3 cloves | | Once a day | |  |  |
|  |  |  |  |  |  | Bulb | *Sinda* | Pound with *A. calamus, J. gendarussa, V. unguiculata, P. betle, Z. officinale* and *C. longa* then rub extract on the body | 3 cloves | | Once or thrice a day | |  |  |
| *Allium fistulosum* L. HNUL 0021401 | Sibuyas dahon | Amaryllidaceae | 0.04 | 0.04 | 0.08 | Leaf | Teething syndrome | Chew leaf | 1 leaf | | Once a day | | Bad breath |  |
| *Mangifera indica* L. HNUL 0021422 | Paho | Anacardiaceae | 0.07 | 0.05 | 0.23 | Bark | Stomachache | Scrape inner bark and boil with *P. americana* and *T. indica* then drink decoction | Hand- length bark | | Serve like water | | None |  |
|  |  |  |  |  |  | Bark | Cough | Scrape inner bark and boil then drink decoction | Hand-length of bark | | Serve like water | |  |  |
|  |  |  |  |  |  | Leaf | Kidney trouble, *doklong* | Boil with *S. koetjape*, *A. buniu*s, *A. muricata*, *A. carambola*, A*. bilimbi*, and *C. maxima* then drink and bath decoction | 7 leaves | | Once a day | |  |  |
| *Spondias pinnata* (L.f.) Kurz HNUL 0021393 | Alobihod | Anacardiaceae | 0.03 | 0.03 | 0.07 | Bark | Cough | Scrape inner bark then crush and drink extract | 2 spoonsful of extract | | Thrice a day | | None |  |
| *Annona muricata* L. HNUL 0021327 | Babana | Annonaceae | 0.56 | 0.39 | 0.88 | Leaf | Urinary tract infection (UTI) | Drink decoction; soak young leaves in warm water and drink | 5 or 7 leaves | | Thrice a day | | None |  |
|  |  |  |  |  |  | Leaf, fruit | Cancer | Drink leaf decoction; eat fruit | 7 leaves; medium sliced fruit | | Thrice a day | |  |  |
|  |  |  |  |  |  | Leaf | Hypertension | Drink decoction | 7 leaves | | Thrice a day | |  |  |
|  |  |  |  |  |  | Leaf | High uric acid | Drink decoction | 7 leaves | | Thrice a day | |  |  |
|  |  |  |  |  |  | Leaf | Pneumonia | Drink decoction | 7 leaves | | Thrice a day | |  |  |
|  |  |  |  |  |  | Leaf | Dizziness | Apply on the forehead | 7 leaves | | Thrice a day | |  |  |
|  |  |  |  |  |  | Leaf | Intestinal cleansing | Drink decoction | 7 leaves | | Twice a day | |  |  |
|  |  |  |  |  |  | Leaf | Kidney trouble, *doklong* | Soak in warm water and drink; boil with *S. koetjape*, *M. indica*, *A. bunius*, *A. carambola*, *A. bilimbi*, and *C. maxima* then drink and bath decoction | 7 leaves | | Once a day | |  |  |
|  |  |  |  |  |  | Leaf | Itchy throat | Apply young leaves on the throat | 7 leaves | | Once a day | |  |  |
|  |  |  |  |  |  | Fruit | Amoebiasis | Eat fruit | Medium sliced fruit | | Thrice a day | |  |  |
|  |  |  |  |  |  | Fruit | Lump, rheumatoid arthritis | Scrape the mesocarp of a young fruit and apply | 1 small sized fruit | | Thrice a day | |  |  |
| *Annona squamosa* L. HNUL 0021404 | Atis | Annonaceae | 0.12 | 0.11 | 0.33 | Leaf | Fever | Drink decoction | 7 leaves | | Thrice a day | | None |  |
|  |  |  |  |  |  | Leaf | Cough, *doklong* | Boil alone or with *C. maxima*, *C. microcarpa*, *A. carambola*, *A. bilimbi*, *T. indica*, *S. koetjape*, *S. cumini*, and *C. nucifera*'s vinegar then drink decoction | 7 leaves | | Once or thrice day | |  |  |
|  |  |  |  |  |  | Leaf | Stomachache | Drink decoction | 7 leaves | | Serve like water | |  |  |
|  |  |  |  |  |  | Leaf | Postpartum care and recovery | Boil alone or with *C. carambola*, *C. bilimbi*, *C. maxima* or with *I. cylindrica*, *J. gendarussa*, and *A. bunius* then drink one glass and the rest is for body steaming and bathing | 7 leaves | | To be performed nine days after giving birth | |  |  |
| *Centella asiatica* (L.) Urb. HNUL 0021423 | Yahong-yahong | Apiaceae | 0.05 | 0.05 | 0.19 | Leaf, stem | Stomachache | Drink leaf decoction; Boil stolon with *M. pudica* and drink decoction | Handful of leaves or stem | | Thrice a day | | None |  |
|  |  |  |  |  |  | Leaf, stem | UTI | Boil leaf or stolon with *I. cylindrica* and drink decoction | Handful of leaves or stem | | Thrice a day | |  |  |
|  |  |  |  |  |  | Leaf | Cough | Boil or soak in water then drink | 7 leaves | | Thrice a day | |  |  |
| *Alstonia scholaris* (L.) R.Br. HNUL 0021361 | Bita | Apocynaceae | 0.27 | 0.03 | 0.07 | Bark | Postpartum care and recovery | Boil alone or with *B. balsamifera, C. maxima, B. spinosa,* *C. sumatranum*, *C. citratus*, *M. balbisiana* cv*.*, *E. indica*, *C. nucifera,* and beeswax then drink two glasses of decoction and bath the rest | 7 leaves | | Thrice a day | | Can cause miscarriage in pregnant woman if decoction is taken orally |  |
| *Catharanthus roseus* (L.) G.Don HNUL 0021311 | Rosas de baybayon | Apocynaceae | 0.15 | 0.12 | 0.39 | Whole plant | Typhoid fever | Drink decoction | 1 whole plant | | Serve like water | | Can cause miscarriage in pregnant |  |
|  |  |  |  |  |  | Leaf | Diabetes; hypertension | Drink decoction | 7 leaves | | Serve like water | |  |  |
|  |  |  |  |  |  | Leaf, flower, root | UTI | Drink leaf, flower, or root decoction | 7 leaves, 7 flowers, handful of roots | | Serve like water | |  |  |
|  |  |  |  |  |  | Leaf | Kidney problem, diarrhea, | Drink decoction | 7 leaves | | Serve like water | |  |  |
|  |  |  |  |  |  | Leaf | Cancer | Drink decoction | 7 leaves | | Serve like water | |  |  |
| *Tabernaemontana pandacaqui* Poir. HNUL 0021416 | Alibotbot | Apocynaceae | 0.05 | 0.05 | 0.1 | Sap, leaf | Boils | Apply sap on the affected area; pound leaves and apply | 7 leaves | | Thrice a day | | None |  |
| *Alocasia macrorrhizos* (L.) G.Don HNUL 0021417 | Badyang | Araceae | 0.08 | 0.08 | 0.12 | Petiole | Tooth decay | Pound the decaying petiole with *C. nucifera'*s matured endosperm then wrap in a black cloth and rub on the jaw area | 1 petiole | | Once a day | | None |  |
| *Alocasia* cultivar HNUL 0021418 | Badyang (itom) | Araceae | 0.08 | 0.08 | 0.17 | Leaf | Stomachache, abdominal pain | Apply on the stomach or on the abdomen | 1 leaf | | Twice a day | | None |  |
|  |  |  |  |  |  | Leaf | *Inaswang* | Heat and apply on the stomach | 1 leaf | | Twice a day (morning and noon) | |  | |
| *Colocasia esculenta* (L.) Schott HNUL 0021424 | Dagmay | Araceae | 0.03 | 0.03 | 0.12 | Petiole | Athlete's foot | Heat and apply | 1 petiole | | Once a day | | None | |
|  |  |  |  |  |  | Leaf | Breast engorgement | Apply on the breast | 1 leaf | | Thrice a day | |  | |
| *Homalomena philippinensis* Engl. HNUL 0021320 | Talos | Araceae | 0.16 | 0.12 | 0.3 | Leaf | Headache, dizziness | Apply on the forehead | 1 leaf | | Once a day (day or night) | | None | |
|  |  |  |  |  |  | Leaf | Abdominal pain | Appy on the abdomen | 1 leaf | | Once a day | |  | |
|  |  |  |  |  |  | Rhizome | Boils | Slice rhizome and apply on the affected area | 1 rhizome | | Once a day | |  | |
|  |  |  |  |  |  | Rhizome | Splinter | Heat and apply on the affected area | 1 rhizome | | Once a day | |  | |
| *Schefflera elliptica* (Blume) Harms HNUL 0021394 | Kalangkang | Araliaceae | 0.04 | 0.04 | 0.08 | Leaf | Postpartum care and recovery | Boil alone or with *E. indica,* *B. spinosa,* and *O. sativa* or *I. cylindrica* drink 1 glass and the rest is for body steaming and bathing | 7 compound leaves | | To be performed nine days after giving birth | | None | |
| *Areca catechu* L. HNUL 0021360 | Bunga | Arecaceae | 0.04 | 0.04 | 0.08 | Seed | Bloated stomach | Wrap a pinch of nut seeds in a *P. betle* leaf daubed with slaked lime (*apog*) then chew or pound and rub extract on the stomach | Pinch of seeds | | Once a day | | Discoloration (red) of teeth and gums | |
| *Cocos nucifera* L. HNUL 0021408 | Niyog | Arecaceae | 0.23 | 0.21 | 0.53 | Fruit | UTI | Drink water of young fruit | 1 fruit | | Once a day | | None | |
|  |  |  |  |  |  | Fruit | Stomachache | Process into oil and rub on the stomach | 1 fruit | | Once or twice | |  | |
|  |  |  |  |  |  | Fruit | Diarrhea | Eat immature endosperm | 2 fruits | | Once a day | |  | |
|  |  |  |  |  |  | Fruit | Postpartum care and recovery | Boil husk with *B*. *spinosa*, *E. indica*, *B. balsamifera*, *C. maxima*, *A. scholaris*, *C. sumatranum*, *C. citratus*, and *M. balbisiana* cv*.* then drink 1 glass and the rest is for body steaming and bathing. | Palm-sized husk | | To be performed nine days after giving birth | |  | |
|  |  |  |  |  |  | Fruit, leaf | Skin lesion | Pound grilled mature endosperm then apply; burn leaf (used during Palm Sunday) and apply ash | Palm-sized endosperm; 1 leaf | | Once a day | |  | |
|  |  |  |  |  |  | Flower | Typhus | Add vinegar (inflorescence sap) in pounded *C. longa* then rub extract onto the head | Half cup vinegar | | Once a day | |  | |
|  |  |  |  |  |  | Flower | Typhoid fever | Rub vinegar (inflorescence sap) on the body | 3-5 spoons | | Once a day | |  | |
|  |  |  |  |  |  | Flower | Fever | Rub vinegar (inflorescence sap) on the body; pound *C. longa* and *Z. officinale* then add vinegar and rub on the head and stomach | 3-5 spoons | | Once a day | |  | |
|  |  |  |  |  |  | Flower | Cough | Process inflorescence sap into vinegar then boil with *A. squamosa*, *C. maxima*, *A. carambola*, *A. bilimbi*, *S. cumini*, *S. koetjape*, and *C. microcarpa* then drink decoction | Half cup vinegar | | Thrice a day | |  | |
|  |  |  |  |  |  | Root | Postpartum bleeding, abdominal pain | Drink decoction | 7 hand-length roots | | Serve like water | |  | |
| *Corypha utan* Lam. HNUL 0021366 | Buri | Arecaceae | 0.03 | 0.03 | 0.12 | Petiole | Limb pain | Boil with *C. citratus* and apply after massaging the foot | 1-foot sliced petiole | | Once | | None | |
|  |  |  |  |  |  | Leaf | Cramps | Tie a strip of leaf around the affected leg | 1 leaf | | Once | |  | |
| *Cordyline fruticosa* (L.) A.Chev. HNUL 0021388 | Baston ni San Hosep | Asparagaceae | 0.03 | 0.03 | 0.07 | Leaf, shoot | Headache | Apply leaves on the forehead; pound heated shoots and rub extract on the body | 3 or 4 leaves; 3 shoots | | Twice a day | | None | |
| *Aloe vera* (L.) Burm.f. HNUL 0021356 | Alobera | Asphodelaceae | 0.04 | 0.04 | 0.13 | Leaf | Burn | Apply succulent | 1 large leaf | | Once a day | | None | |
|  |  |  |  |  |  | Leaf | Dandruff | Rub succulent on the head | 3 leaves | | Once a day | |  | |
| *Artemisia vulgaris* L. HNUL 0021309 | Herba/Artemisa | Asteraceae | 0.59 | 0.57 | 0.77 | Leaf | Cough | Crush and rub extract on the head, body or back; crush heated leaves with salt and rub extract; crush and drink extract alone or with breastmilk | Handful of leaves | | Once a day (night) or twice a day | | None | |
|  |  |  |  |  |  | Leaf | Muscle pain, chest pain | *To-om* and rub extract on the body; crush and rub extract | Handful of leaves | | Once a day (night) | |  | |
|  |  |  |  |  |  | Leaf | Fever, headache | *To-om* and rub extract on the body; crush and rub or drink extract; Boil and drink 1 glass of decoction then bath the rest | Handful of leaves | | Rubbing of extract is done twice or thrice a day; bathing of decoction is done once a day | |  | |
|  |  |  |  |  |  | Leaf | Fracture | Crush and apply extract on the affected area | Handful of leaves | | Thrice a day | |  | |
|  |  |  |  |  |  | Leaf | Hearing impairment | Heat and apply on the mastoid area | 5 leaves | | Thrice a day | |  | |
| *Bidens pilosa* L. HNUL 0021337 | Tubok-tubok | Asteraceae | 0.07 | 0.05 | 0.14 | Sap, root | Tooth decay | Apply sap on the jaw area; pound root and insert in the decaying tooth | Generous amount of sap; 1 root | | Once a day | | None | |
|  |  |  | 0 |  |  | Leaf | Cuts, wounds | Crush and apply extract | 7 leaves | | Once a day | |  | |
| *Blumea balsamifera* (L.) DC. HNUL 0021339 | Alibhon | Asteraceae | 0.64 | 0.44 | 0.8 | Leaf, root | Cough | Crush leaves and drink extract; eat young leaves; crush leaves and rub on the head; drink root or leaf decoction | 2 spoonsful of extract; 3 young leaves | | Thrice a day | | None | |
|  |  |  |  |  |  | Leaf | Headache | Apply alone or with *J. gendarussa*, *C. longa*, and *Z. officinale* on the forehead; crush and apply extract on the body | 7 leaves | | Once a day | |  | |
|  |  |  |  |  |  | Leaf | Muscle pain | Pound with *C. citratus* and rub extract on the body | 7 leaves | | Thrice a day | |  | |
|  |  |  |  |  |  | Leaf | Vomiting blood | Eat young leaves | 3 leaves | | Thrice a day | |  | |
|  |  |  |  |  |  | Leaf | Bloated stomach/gas pain, *inaswang* | Mix with *J. gendarussa*, *Z. officinale*, *C. longa,* chicken's blood then after an hour apply on the stomach; apply with *C. sappan*, *J. gendarussa*, and *C. maxima* on the stomach after offering some discreet prayer(oration) | 7 leaves | | Once | |  | |
|  |  |  |  |  |  | Leaf | Goiter, UTI | Drink decoction | 7 leaves | | Thrice a day | |  | |
|  |  |  |  |  |  | Leaf | Postpartum care and recovery | Boil alone or with *F. benjamina*, *C. sumatranum*, *C. maxima*, *J. gendarussa*, *B*. *spinosa* or with *I. cylindrica* for body steaming and bathing; boil with *C. maxima*, *B*. *spinosa*, *C. sumatranum*, *A. scholaris*, *C. citratus*, *M. balbisiana* cv*.*, *E. indica*, *C. nucifera,* beeswax*,* or with *Z. officinale* then drink two glasses and the rest is for body steaming and bathing | 7 leaves | | To be performed nine days after giving birth | |  | |
| *Chromolaena odorata* (L.) R.M.King & H.Rob. HNUL 0021306 | Hagonoy | Asteraceae | 0.36 | 0.36 | 0.4 | Leaf | Cuts, wounds | Crush and apply extract | 5 leaves | | Once | | None | |
| *Elephantopus tomentosus* L. HNUL 0021321 | Dila-dila ka baka | Asteraceae | 0.12 | 0.09 | 0.14 | Leaf | Diarrhea | Drink decoction; crush and drink extract | 7 leaves; 2 spoonsful of extract | | Twice a day | | None | |
|  |  |  |  |  |  | Leaf, root | Stomachache | Crush leaves and drink extract, eat young leaves; crush and apply leaves on the stomach; drink root decoction | 2 spoonsful of extract; 3 or 5 young leaves; handful of roots | | Once a day | |  | |
|  |  |  |  |  |  | Leaf | Vomiting blood | Crush and drink extract | 1 spoonful | | Thrice a day | |  | |
| *Impatiens balsamina* L. HNUL 0021318 | Solangga | Balsaminaceae | 0.16 | 0.16 | 0.34 | Flower | Cuts/wounds | Crush and apply extract | 3 or 7 flowers | | Thrice a day | | Can cause miscarriage in pregnant woman | |
|  |  |  |  |  |  | Flower | Boils | Crush and apply extract | 7 flowers | | Thrice a day | |  | |
|  |  |  |  |  |  | Flower | Lump | Crush and apply extract | 7 flowers | | Thrice a day | |  | |
|  |  |  |  |  |  | Leaf, flower | Birth control | Crush leaves or flowers then soak in water and drink | 7 flowers or leaves | | Thrice a day | |  | |
| *Basella alba* L. HNUL 0021355 | Alugbati | Basellaceae | 0.19 | 0.17 | 0.22 | Leaf | Boils | Apply softened leaf on the affected area | 1 leaf | | Thrice a day | | None | |
|  |  |  |  |  |  | Leaf | Mumps | Apply softened leaf on the affected area | 1 or 3 leaves | | Thrice a day | |  | |
| *Bixa orellana* L. HNUL 0021302 | Istiwitis | Bixaceae | 0.17 | 0.16 | 0.29 | Leaf | Headache | Apply on the forehead; apply on the forehead and on the stomach area | 5 or 7 leaves | | Twice a day or once a day (afternoon) | | None | |
|  |  |  |  |  |  | Leaf | Dizziness | Apply on the forehead | 5 or 7 leaves | | Twice a day or once a day (afternoon) | |  | |
|  |  |  |  |  |  | Leaf | Bloated stomach/gas pain | Apply fresh or heated leaves on the stomach area | 5 or 7 leaves | | Thrice a day | |  | |
|  |  |  |  |  |  | Seed | Rashes | Apply on the affected area | 7 seeds | | Once or twice a day | |  | |
| *Cordia dichotoma* G.Forst. HNUL 0021390 | Anonang | Boraginaceae | 0.05 | 0.05 | 0.19 | Bark | Fracture | Heat scraped bark then apply | 1 foot bark | | Once a day | | None | |
|  |  |  |  |  |  | Bark | Postpartum care and recovery | Drink decoction | 1 foot bark | | To be performed nine days after giving birth | |  | |
|  |  |  |  |  |  | Leaf | Headache | Crush and drink extract | 7 leaves | | Once a day | |  | |
| *Brassica rapa* L. HNUL 0021403 | Pechay | Brassicaceae | 0.03 | 0.03 | 0.12 | Leaf | Kidney problem | Crush and drink | 3 leaves | | Once a day | | None | |
|  |  |  |  |  |  | Leaf | Anemia | Cook as vegetable and eat | Handful of leaves | | Once a day | |  | |
| *Ananas comosus* (L.) Merr. HNUL 0021431 | Pinya | Bromeliaceae | 0.04 | 0.04 | 0.18 | Stem | Headache | Pound cabbage and apply on the forehead | 1 stem | | Once a day | | None | |
|  |  |  |  |  |  | Root | Hypertension | Drink decoction | Roots of 1 pineapple plant | | Thrice day | |  | |
|  |  |  |  |  |  | Root | Bleeding gums | Drink decoction | Roots of 1 pineapple plant | | Twice a day | |  | |
| *Carica papay*a L. HNUL 0021410 | Kapayas (laki) | Caricaceae | 0.35 | 0.33 | 0.6 | Stem | Typhoid fever | Scrape inner stem (male plant) and apply on the forehead; apply on the stomach and forehead | Handful of scraped stems | | Once or twice a day | | None | |
|  |  |  |  |  |  | Stem | Dengue | Scrape inner stem (male plant) then crush and drink extract; pound and drink extract | Handful of scraped stems | | Once or twice a day | |  | |
|  |  |  |  |  |  | Stem | Headache | Scrape stem (male plant) and apply of the stomach and forehead | Handful of scraped stems | | Once or twice a day | |  | |
|  |  |  |  |  |  | Stem | Fever | Scrape stem (male plant) then crush and drink extract | Handful of scraped stems | | Once or twice a day | |  | |
|  |  |  |  |  |  | Stem | *Doklong* | Scrape and apply | Handful of scraped stems | | Once or twice a day | |  | |
|  |  |  |  |  |  | Fruit | Lactation support | Eat ripe fruit | 1 small-size fruit | | Once a day | |  | |
|  |  |  |  |  |  | Fruit | Constipation | Eat ripe fruit | 1 small-size fruit | | Once a day | |  | |
|  |  |  |  |  |  | Fruit | Amoebiasis | Eat ripe fruit | 1 small-size fruit | | Once a day | |  | |
| *Ipomoea batatas* (L.) Lam. HNUL 0021407 | Kamote | Convolvulaceae | 0.03 | 0.03 | 0.12 | Leaf | Breast engorgement | Crush heated leaves and apply on the breast | Handful of leaves | | Thrice a day | | None | |
|  |  |  |  |  |  | Leaf | Anemia | Blanch leaves and eat | Handful of leaves | | Once a day | |  | |
| *Decalobanthus peltatus* (L.) A.R.Simões & Staples | Burakan | Convolvulaceae | 0.08 | 0.08 | 0.22 | Leaf | Muscle swelling | Apply on the affected area | 1 leaf | | Once a day (night) | | None | |
|  |  |  |  |  |  | Leaf | Headache | Apply on the forehead | 1 leaf | | Twice a day | |  | |
|  |  |  |  |  |  | Fruit | Swelling of male genitalia | Pound and apply | 1 fruit | | Thrice a day | |  | |
|  |  |  |  |  |  | Leaf | Rheumatoid arthritis | Apply on the legs | 1 leaf | | Thrice a day | |  | |
| *Cheilocostus speciosus* (J.Koenig) C.D.Specht HNUL 0021333 | Tabungyan | Costaceae | 0.01 | 0.01 | 0.05 | Stem | Headache | Pound alone or with *E. philippinensis, Amomum* sp., *L. circinnatum, C. longa,* and *Z. officinale* then rub extract on the head | 1 stem | | Once a day | | None | |
| *Kalanchoe pinnata* (Lam.) Pers. HNUL 0021374 | Maritana | Crassulaceae | 0.13 | 0.13 | 0.22 | Leaf | Boils | Apply on the affected area | 1 leaf | | Once a day | | None | |
|  |  |  |  |  |  | Leaf | Mumps | Apply on the affected area | 1 leaf | | Once a day | |  | |
|  |  |  |  |  |  | Leaf | Toothache | Crush and apply on the jaw area | 1 leaf | | Once a day | |  | |
| *Cucurbita maxima* Duchesne HNUL 0021420 | Kurbasa | Cucurbitaceae | | 0.03 | 0.16 | Tendrils | Fever | Pound with *V. unguiculata* and rub extract on the body | Handful of tendrils | | Once a day | | None | |
|  |  |  |  |  |  | Tendrils | Stomachache, swelling in female genitalia | Pound and apply | Handful of tendrils | | Once a day | |  | |
| *Luffa aegyptiaca* Mill. HNUL 0021399 | Patola | Cucurbitaceae | | 0.01 | 0.06 | Fruit | Hypertension | Cook as vegetable and eat | 1 fruit | | Once a day | | None | |
| *Momordica charantia* L. HNUL 0021313 | Amargoso | Cucurbitaceae | 0.25 | 0.25 | 0.43 | Leaf | UTI | Boil with *M. oleifera, Cissus* sp., and *C. citratus* then drink decoction | 7 leaves | | Thrice a day | | None | |
|  |  |  |  |  |  | Leaf | Cough | Crush and drink extract alone or with sugar | 1 spoonful or 1/2 spoon of extract | | Once a day | |  | |
|  |  |  |  |  |  | Leaf | Anemia | Crush and drink extract | Handful of leaves | | Once a day | |  | |
|  |  |  |  |  |  | Leaf | Cuts, wounds | Crush and apply | Handful of leaves | | Thrice a day | |  | |
| *Cyperus mindorensis* (Steud.) Huygh HNUL 0021395 | Butonsilyo | Cyperaceae | 0.07 | 0.05 | 0.23 | Whole plant | Fever | Drink decoction | 1 whole plant | | Thrice a day | | None | |
|  |  |  |  |  |  | Whole plant | Headache | Drink decoction | 1 whole plant | | Thrice a day | |  | |
|  |  |  |  |  |  | Root | Stomachache | Boil with *E. indica* and drink decoction | Roots of 1 whole plant | | Thrice a day | |  | |
|  |  |  |  |  |  | Whole plant | Measles | Drink decoction | 1 whole plant | | Thrice a day | |  | |
| *Dioscorea esculenta* (Lour.) Burkill HNUL 0021402 | Tam-is | Dioscoreaceae | 0.07 | 0.07 | 0.11 | Tuber | Shingles | Slice tuber in half and rub on the affected area | 1 tuber | | Three, four or five times | | None | |
|  |  |  |  |  |  | Tuber | Mumps | Grate and apply | 1 tuber | | Twice a day | |  | |
|  |  |  |  |  |  | Tuber | Ringworm | Slice tuber in half and rub on the affected area | 1 tuber | | Once a day | |  | |
| *Euphorbia hirta* L. HNUL 0021305 | Tawa-tawa | Euphorbiaceae | 0.27 | 0.23 | 0.36 | Whole plant, root | Typhoid fever | Drink decoction | 7 whole plants; handful of roots | | Thrice a day | | None | |
|  |  |  |  |  |  | Whole plant | Dengue | Drink decoction | 7 whole plants | | Serve like water | |  | |
|  |  |  |  |  |  | Whole plant, root | UTI | Drink decoction | 7 whole plant, handful of roots | | Serve like water | |  | |
|  |  |  |  |  |  | Sap | Angular cheilitis | Apply sap on the affected area | Sap from 1 stem | | Apply as needed | |  | |
| *Euphorbia tirucalli* L. HNUL 0021325 | Bari-bari/tul-an-tulan | Euphorbiaceae | 0.03 | 0.01 | 0.1 | Sap | Boils | Apply sap on the affected area | Palm-length stem | | Apply as needed | | None | |
|  |  |  |  |  |  | Sap | Toothache/tooth decay | Drop sap on the affected tooth | Palm-length stem | | Apply as needed | |  | |
| *Jatropha curcas* L. HNUL 0021368 | Kasla | Euphorbiaceae | 0.55 | 0.36 | 0.72 | Leaf | Headache, chill | Apply on the forehead | 7 leaves | | Thrice a day | | None | |
|  |  |  |  |  |  | Leaf, bark | Fracture | Apply leaves on the affected area; scrape bark and apply; heat scrape bark and apply | 3 or 5 leaves; 1 foot bark | | Twice a day | |  | |
|  |  |  |  |  |  | Leaf | Spasm | Apply on the affected area | 7 leaves | | Twice a day | |  | |
|  |  |  |  |  |  | Bark | Cuts, wounds | Scrape inner bark and apply on the affected area | Handful of scraped bark | | Twice a day | |  | |
|  |  |  |  |  |  | Bark | *Kolebra* | Scrape inner bark then crush and drink extract | 1 foot bark | | Twice a day | |  | |
|  |  |  |  |  |  | Bark | Tetanus | Scrape inner bark then *to-om* and apply | 2-foot bark | | Twice a day | |  | |
|  |  |  |  |  |  | Sap | Oral thrush | Drop sap on the tongue | Sap from 3 petioles | | Once a day | |  | |
|  |  |  |  |  |  | Leaf, bark | Diarrhea | Drink leaf or bark decoction | 7 leaves | | Thrice a day | |  | |
|  |  |  |  |  |  | Leaf | Stomachache | Drink decoction; apply on the stomach | 5 or 7 leaves | | Thrice a day | |  | |
|  |  |  |  |  |  | Leaf | Gas pain | Apply fresh or heated leaves on the stomach area | 6 or 7 leaves | | Thrice a day | |  | |
|  |  |  |  |  |  | Leaf, bark | Muscle pain*,* animal (pig) bite | Apply on the affected area | 1 or 3 leaves | | Thrice a day | |  | |
| *Manihot esculenta* Crantz HNUL 0021377 | Balinghoy | Euphorbiaceae | 0.08 | 0.08 | 0.26 | Tuber | Shingles | Grate and apply on the affected area | 1 small tuber | | Thrice a day | | None | |
|  |  |  |  |  |  | Leaf | Fracture | Apply on the affected area | 3 or 5 leaves | | Thrice a day | |  | |
|  |  |  |  |  |  | Leaf | Gas pain | Heat and apply on the stomach | 7 leaves | | Thrice a day | |  | |
|  |  |  |  |  |  | Leaf | Lower back pain | Apply on the lower back | 3 leaves | | Twice a day | |  | |
| *Caesalpinia sappan* L. HNUL 0021392 | Sibukaw | Fabaceae | 0.11 | 0.09 | 0.32 | Bark | Typhoid fever | Scrape inner bark add *C. nucifera*'s vinegar then crush and rub extract on the head | Handful of scraped bark | | Twice a day | | None | |
|  |  |  |  |  |  | Bark | Fever | Scrape inner bark then crush and drink extract; pound with *C. longa* and *Z. officinale* then drink some extract and rub on the pulse points | Handful of scraped bark | | Once a day | |  | |
|  |  |  |  |  |  | Bark | Stomachache | Scrape inner bark then crush and drink extract | Handful of scraped bark | | Once a day | |  | |
|  |  |  |  |  |  | Bark | UTI | Drink decoction | Handful of scraped bark | | Thrice a day | |  | |
|  |  |  |  |  |  | Leaf | *Inaswang* | Apply with *B. balsamifera*, *J. gendarussa*, *C. maxima* on the stomach area (with discreet oration) | 7 compound leaves | | Once a day | |  | |
| *Cajanus cajan* (L.) Huth HNUL 0021316 | Kadyos | Fabaceae | 0.11 | 0.11 | 0.15 | Leaf | Stomachache | Pound and drink pure extract or add sugar; drink decoction | 7 leaves | | Twice a day | | None | |
|  |  |  |  |  |  | Leaf | Diarrhea | Pound and drink pure extract or add sugar; drink decoction | 7 leaves | | Thrice a day | |  | |
|  |  |  |  |  |  | Leaf | Vomiting blood | Drink decoction | 7 leaves | | Serve like water | |  | |
| *Clitoria ternatea* L. HNUL 0021358 | Blue ternate | Fabaceae | 0.04 | 0.03 | 0.12 | Flower | Cancer | Soak in hot water and drink | 7 flowers | | Thrice a day | | None | |
|  |  |  |  |  |  | Flower | Hypertension | Eat directly; Soak in hot water and drink | 7 flowers | | Thrice a day | |  | |
| *Desmodium triflorum* (L.) DC HNUL 0021425 | Himbis-himbis | Fabaceae | 0.07 | 0.04 | 0.22 | Leaf | Stomachache | Drink decoction | Handful of leaves | | Thrice a day | | None | |
|  |  |  |  |  |  | Leaf | Headache | Drink decoction | Handful of leaves | | Thrice a day | |  | |
|  |  |  |  |  |  | Leaf | Measles | Drink decoction | Handful of leaves | | Thrice a day | |  | |
|  |  |  |  |  |  | Whole plant | Kidney stones, UTI | Boil with *I. cylindrica* and *B. spinosa* then drink decoction | Handful of whole plants | | Thrice a day | |  | |
| *Gliricidia sepium (Jacq.)* Kunth ex Walp HNUL 0021303 | Madre de kakaw | Fabaceae | 0.41 | 0.27 | 0.44 | Leaf, bark | Cuts, wounds | Apply leaf extract; scrape bark and apply extract | Handful of scraped bark or leaves | | Once a day | | None | |
|  |  |  |  |  |  | Leaf | Skin lesion | Pound and apply extract on the affected area | Handful of leaves | | Once a day | |  | |
|  |  |  |  |  |  | Leaf | Stomachache | Apply on the stomach | 7 leaves | | Once a day | |  | |
|  |  |  |  |  |  | Leaf, bark | Pityriasis rosea | Scrape inner bark and apply extract; *to-om* leaves and apply extract | 3 leaves; handful of scraped bark | | Once a day | |  | |
|  |  |  |  |  |  | Leaf | Postpartum bleeding; postpartum care and recovery | Apply fresh or heated leaves on the abdomen; sit on the heated leaves | Handful of leaves | | Once a day | |  | |
|  |  |  |  |  |  | Bark | Fracture | Scrape inner bark and apply | Handful of scraped bark | | Twice a day | |  | |
| *Indigofera tinctoria* L. HNUL 0021362 | Tagum | Fabaceae | 0.04 | 0.04 | 0.08 | Leaf | Diarrhea, stomachache | Burn as incense | Handful of leaves | | Once | | None | |
| *Leucaena leucocephala* (Lam.) de Wit HNUL 0021382 | Agho/Ipil-ipil | Fabaceae | 0.12 | 0.12 | 0.21 | Seed | Helminthiasis | Eat seed; roast mature seeds and eat | Handful of seeds | | Once | | None | |
|  |  |  |  |  |  | Bark | Induce labor | Add raw egg in bark's extract and apply on the stomach | Handful of scraped bark | | Once | |  | |
| *Mimosa pudica* L. HNUL 0021338 | Huya-huya | Fabaceae | 0.11 | 0.11 | 0.33 | Root | Induce period (menstruation) | Drink decoction | Handful of roots | | Once a day | | None | |
|  |  |  |  |  |  | Root | Stomachache | Boil with *C. asiatica* and drink decoction | Roots of 1 whole plant | | Serve like water | |  | |
|  |  |  |  |  |  | Root | UTI, kidney stones | Boil with *Z. mays* silk and drink decoction | Roots of 1 whole plant | | Serve like water | |  | |
|  |  |  |  |  |  | Whole plant | Malaise | Burn as incense for infants and children | 1 whole plant | | Once | |  | |
|  |  |  |  |  |  | Whole plant | Postpartum care and recovery | Burn as incense after giving birth | 1 whole plant | | Once | |  | |
| *Phaseolus lunatus* L. HNUL 0021375 | Patani | Fabaceae | 0.07 | 0.07 | 0.2 | Leaf | Pityriasis rosea | Pound and rub extract | 7 leaves | | Thrice a day | | None | |
|  |  |  |  |  |  | Leaf | Mumps | Pound and apply | 7 leaves | | Thrice a day | |  | |
|  |  |  |  |  |  | Leaf | Bloated stomach | Pound and apply on the stomach | 7 leaves | | Once | |  | |
| *Pithecellobium dulce* (Roxb.) Benth. HNUL 0021413 | Kamunsil | Fabaceae | 0.03 | 0.03 | 0.07 | Leaf | Cough | Drink decoction | Handful of leaves | | Thrice a day | | None | |
| *Senna alata* (L.) Roxb. HNUL 0021353 | Palotsina | Fabaceae | 0.11 | 0.07 | 0.16 | Leaf | Pityriasis versicolor, Pityriasis rosea, ringworm | Crush or pound and apply extract | 7 or 9 leaves | | Once or thrice a day a day | | None | |
| *Tamarindus indica* L. HNUL 0021432 | Sambag | Fabaceae | 0.13 | 0.12 | 0.21 | Leaf | Cough | Crush and drink extract or add with sugar; wash with warm water then crush and drink extract; drink decoction; boil alone or with *A. squamosa, C. maxima, A. carambola, A. bilimbi, S. cumini*, and *S. koetjape* then drink decoction | 1 spoonful of extract; handful of leaves | | Twice a day | |  | |
|  |  |  |  |  |  | Leaf | Headache | Boil with *A. carambola* and bath decoction | Handful leaves | | Once a day | |  | |
| *Vigna unguiculata* (L.) Walp. HNUL 0021411 | Hantak/Latoy | Fabaceae | 0.07 | 0.07 | 0.25 | Seed | Bloated stomach; stomachache | Chew with *O. sativa* and *Z. officinale* then rub on the stomach | 7 seeds | | Once a day | | None | |
|  |  |  |  |  |  | Leaf | Fever | Crush and rub extract on the body; pound with *C. maxima* and rub extract on the body | 7 leaves | | Twice a day | |  | |
|  |  |  |  |  |  | Leaf | Skin lesion | Crush and apply | 7 leaves | | Once a day | |  | |
|  |  |  |  |  |  | Seed | *Sinda* | Pound with *A. calamus*, *J. gendarussa*, *P. betle*, *A. sativum*, and *C. longa* and rub extract on the body | 7 leaves | | Once a day | |  | |
| *Cratoxylum sumatranum* (Jack) Blume HNUL 0021365 | Kansilay | Hypericaceae | 0.09 | 0.09 | 0.18 | Leaf | Postpartum care and recovery | Boil with *B. balsamifera*, *C. maxima*, *J. gendarussa*, and *B. spinosa*, *A. scholaris*, *C. citratus*, *E. indica*, *M. balbisiana* cv., *C. nucifera*, and beeswax drink two glasses and the rest is for body steaming and bathing | 7 leaves | | To be performed nine days after giving birth | | None | |
|  |  |  |  |  |  | Leaf | Diarrhea | Drink decoction | 7 leaves | | Thrice a day | |  | |
|  |  |  |  |  |  | Bark | Gastric ulcer | Drink decoction | 7 leaves | | Thrice a day | |  | |
| *Eleutherine palmifolia* (L.) Merr. HNUL 0021379 | Sibuyas costa | Iridaceae | 0.05 | 0.05 | 0.14 | Bulb | Rashes | Pound and apply; rub on the affected area | 3 bulbs | | Thrice a day | | None | |
|  |  |  |  |  |  | Bulb | Headache | Crush in hot water and rub extract into the head and body | 3 bulbs | | Thrice a day | |  | |
| *Clerodendrum quadriloculare* (Blanco) Merr. HNUL 0021419 | Salin-uwak | Lamiaceae | 0.03 | 0.03 | 0.07 | Leaf | Headache | Apply on the forehead | 3 leaves | | Thrice a day | | None | |
| *Gmelina arborea* Roxb. ex Sm*.* HNUL 0021330 | Gimelina | Lamiaceae | 0.27 | 0.17 | 0.4 | Leaf | Muscle swelling, limb pain, lower back pain | Apply young leaves on the affected area | 3 leaves | | Thrice a day | | None | |
|  |  |  |  |  |  | Leaf | Headache | Apply on the forehead | 1, 3, 5, or 7 leaves | | Twice a day | | None | |
|  |  |  |  |  |  | Leaf | Dizziness | Apply on the forehead | 3 leaves | | Twice a day | |  | |
|  |  |  |  |  |  | Leaf | Rheumatoid arthritis | Apply on the affected area | 5 or 7 leaves | | Twice a day | |  | |
|  |  |  |  |  |  | Leaf | Stomachache | Apply on the stomach | 3 leaves | | Twice a day | |  | |
|  |  |  |  |  |  | Leaf | Cough | Apply on the back | 3 leaves | | Twice a day | |  | |
|  |  |  |  |  |  | Leaf | Cuts, wounds, splinter | Crush heated fruit and apply extract | 3 leaves | | Twice a day | |  | |
| *Hyptis capitata* Jacq. HNUL 0021308 | Sampok-sampok | Lamiaceae | 0.13 | 0.09 | 0.23 | Leaf | Diarrhea | Apply on the stomach area | Handful of leaves | | Thrice a day | | None | |
|  |  |  |  |  |  | Leaf | Stomachache | *To-om* and apply on the stomach; crush and rub extract on the stomach area; drink decoction | Handful of leaves | | Thrice a day; water replacement | |  | |
|  |  |  |  |  |  | Leaf | Bloated stomach | *To-om* and apply on the stomach | Handful of leaves | | Handful of leaves | |  | |
|  |  |  |  |  |  | Leaf | Helminthiasis | Apply heated leaves on the stomach | Handful of leaves | | Once a day | |  | |
|  |  |  |  |  |  | Leaf | Cuts, wounds | Crush and apply extract | Handful of leaves | | Thrice a day | |  | |
| *Mentha arvensis* L. HNUL 0021357 | Viks | Lamiaceae | 0.03 | 0.03 | 0.07 | Leaf | Rhinorrhea | Crush and sniff | 7 leaves | | Thrice a day | | None | |
| *Orthosiphon aristatus* (Blume) Miq. HNUL 0021332 | Renalin | Lamiaceae | 0.07 | 0.05 | 0.23 | Leaf | UTI | Drink decoction | Handful of leaves | | Thrice a day | |  | |
|  |  |  |  |  |  | Leaf | Cuts, wounds | Crush and apply extract | Handful of leaves | | Twice a day | |  | |
|  |  |  |  |  |  | Leaf | Cancer, kidney stones | Drink decoction | Handful of leaves | | Serve like water | |  | |
| *Plectranthus amboinicus* (Lour.) Spreng. HNUL 0021319 | Oregano | Lamiaceae | 0.32 | 0.31 | 0.44 | Leaf | Cough | Crush fresh or heated leaves and drink extract; crush in hot water and drink; add sugar in extract for infants/children and drink; drink leaf decoction | 1 spoonful of extract; handful of leaves | | Once or twice or thrice a day | | None | |
|  |  |  |  |  |  | Leaf | Cuts, wounds | Crush and apply extract | 7 leaves | | Once a day | |  | |
|  |  |  |  |  |  | Leaf | Boils | Crush and apply | 3 leaves | | Once a day | |  | |
| *Plectranthus scutellarioides* (L.) R.Br. HNUL 0021310 | Rapunaya | Lamiaceae | 0.49 | 0.4 | 0.62 | Leaf | Cough | Crush and drink extract; add sugar in extract and drink | 5 leaves (1 spoonful) | | Twice or thrice a day | | None | |
|  |  |  |  |  |  | Leaf | Tuberculosis | Crush and drink extract | 7 leaves | | Once a day | |  | |
|  |  |  |  |  |  | Leaf | Lump, bruise | Crush and apply on the affected area | 7 leaves | | Once a day | |  | |
|  |  |  |  |  |  | Leaf | Bruise | Crush and apply on the affected area | 7 leaves | | Once a day | |  | |
|  |  |  |  |  |  | Leaf | Fracture | Crush and apply on the affected area | Handful of leaves | | Once a day | |  | |
|  |  |  |  |  |  | Leaf | Muscle pain | Add egg white in leaves extract to be taken orally by infants and children | Handful of leaves | | Once a day | |  | |
|  |  |  |  |  |  | Leaf | Cuts, wounds | Crush and apply extract | 7 leaves | | Once a day | |  | |
| *Tectona grandis* L.f. HNUL 0021397 | Mikrokrom | Lamiaceae | 0.08 | 0.08 | 0.17 | Leaf | Cuts, wounds | Crush leaf and apply extract; scrape bark and apply extract | 1 leaf | | Once a day | | None | |
|  |  |  |  |  |  | Leaf | Rheumatoid arthritis | Apply leaf | 1 leaf | | Once a day | |  | |
| *Vitex trifolia* L. HNUL 0021372 | Lagundi | Lamiaceae | 0.08 | 0.08 | 0.17 | Leaf | Cough | Drink decoction; crush and rub extract | Handful of leaves | | Thrice a day | | None | |
|  |  |  |  |  |  | Leaf | *Doklong* | Drink decoction | Handful of leaves | | Serve like water | |  | |
| *Persea americana* Mill. HNUL 0021426 | Abokado | Lauraceae | 0.04 | 0.04 | 0.13 | Bark, root | Stomachache | Boil root or inner bark with *M. indica* and *T. indica* or with *C. cainito* then drink decoction | Handful of scraped bark or root | | Serve like water | | None | |
|  |  |  |  |  |  | Leaf | Kidney stones | Drink decoction | Handful of leaves | | Serve like water | |  | |
| *Barringtonia asiatica* (L.) Kurz HNUL 0021427 | Biribito-on | Lecythidaceae | 0.03 | 0.03 | 0.12 | Fruit | Lump | Scrape mesocarp and apply | 1 fruit | | Once a day | | None | |
|  |  |  |  |  |  | Fruit | Swelling of male genitalia | Scrape mesocarp and apply | 1 fruit | | Once a day | |  | |
| *Lygodium circinnatum* (Burm. f.) Sw. HNUL 0021322 | Nito | Lygodiaceae | 0.01 | 0.03 | 0.07 | Stem | Headache | Pound with *C. longa*, *Z. officinale*, *C. speciosus*, and *E. philippinensis* then rub extract on the body | 1 meter of vine (stem) | | Once a day | | None | |
| *Lagerstroemia speciosa* (L.) Pers. HNUL 0021335 | Banaba | Lythraceae | 0.13 | 0.09 | 0.23 | Leaf, bark | UTI | Drink decoction | 7 leaves | | Thrice a day | | None | |
|  |  |  |  |  |  | Leaf | Kidney stones | Boil alone or with *Z. may*'s silk then drink decoction | 7 leaves | | Thrice a day | |  | |
|  |  |  |  |  |  | Bark | Cancer | Drink decoction | Handful of scraped bark | | Thrice a day | |  | |
|  |  |  |  |  |  | Bark | *Doklong* | Drink decoction | Handful of scraped bark | | Thrice a day | |  | |
| *Abelmoschus esculentus* (L.) Moench HNUL 0021315 | Okra | Malvaceae | 0.04 | 0.04 | 0.18 | Fruit | UTI | Soak in water and drink | 3 sliced fruits | | Serve like water | | None | |
|  |  |  |  |  |  | Fruit | Cancer | Cook as vegetable and eat | 7 fruits | | Once a day | |  | |
|  |  |  |  |  |  | Fruit | Hypertension | Cook as vegetable and eat | 7 fruits | | Once a day | |  | |
| *Corchorus olitorius* L. HNUL 0021381 | Tagabang | Malvaceae | 0.09 | 0.08 | 0.17 | Leaf | Induce labor | Crush and rub extract on the stomach area | Handful of leaves | | Once | | Can cause miscarriage in woman | |
|  |  |  |  |  |  | Leaf | Constipation | Eat cooked leaves | Handful of leaves | | Once | |  | |
|  |  |  |  |  |  | Seed | Birth control | Soak roasted seeds in water and drink | 1 spoonful of seeds | | Once a day | |  | |
| *Hibiscus acetosella* Welw. ex Hiern HNUL 0021370 | Labog | Malvaceae | 0.08 | 0.07 | 0.29 | Leaf | Cuts, wounds | *To-om* and apply on the affected area | Handful of leaves | | Once | | None | |
|  |  |  |  |  |  | Leaf | Boils | *To-om* and apply on the affected area | Handful of leaves | | Once a day | |  | |
|  |  |  |  |  |  | Leaf | Anemia | Eat as vegetable | Handful of leaves | | Once a day | |  | |
|  |  |  |  |  |  | Leaf | Hypertension, diabetes, rhinorrhea | Drink decoction | Handful of leaves | | Thrice a | |  | |
|  |  |  |  |  |  | Leaf | Cough | *To-om* and rub extract | Handful of leaves | | Once a day | |  | |
| *Urena lobata* L. HNUL 0021340 | Dalupang | Malvaceae | 0.13 | 0.13 | 0.18 | Flower | Boils | Crush and apply on the affected area | Handful of leaves | | Thrice a day | | None | |
| *Sandoricum koetjape* (Burm.f.) Merr. HNUL 0021428 | Santol | Meliaceae | 0.09 | 0.08 | 0.26 | Bark | Stomachache | Boil alone or with *P. americana* and *M. indica* then drink decoction | Handful of scraped bark | | Drink as water | | None | |
|  |  |  |  |  |  | Leaf | Cough | Boil alone or with *A. squamosa*, *C. maxima*, *A. carambol*a, *A. bilimbi*, *S. cumini*, and *C. microcarpa* then drink decoction | 7 leaves | | Drink as water | |  | |
|  |  |  |  |  |  | Leaf | Kidney trouble, *doklong* | Boil with *A. bunius*, *M. indica*, *A. muricat*a, *A. carambola*, *A. bilimbi*, and *C. maxima* then drink and bath decoction | 7 leaves | | Thrice a day | |  | |
| *Swietenia mahagoni* (L.) Jacq. HNUL 0021352 | Mahogani | Meliaceae | 0.23 | 0.16 | 0.25 | Seed, leaf, bark | Stomachache | Drink leaf or bark decoction, eat seed, soak seed in warm water and drink; apply leaves on the stomach area | 7 seeds; 7 leaves; handful of scraped inner bark | | Once or thrice a day | | Can cause miscarriage in pregnant woman | |
|  |  |  |  |  |  | Seed | Birth control | Eat seed; drink decoction | 3 or 7 seeds | | Twice a day | |  | |
|  |  |  |  |  |  | Bark, seed | diarrhea | Drink bark decoction; eat seed | 3 seeds; handful of scraped inner bark | | Twice a day | |  | |
| *Tinospora crispa* (L.) Hook. f. & Thomson HNUL 0021380 | Manunggal | Menispermaceae | 0.21 | 0.16 | 0.39 | Leaf | *Sinda* | Apply with *C. longa*, *Z. officinal*e, *J. gendarussa*, and *P. carruthersii* on the forehead | 1 or 3 leaves | | Twice a day | | Can cause miscarriage in pregnant woman | |
|  |  |  |  |  |  | Bark, sap | Toothache | Scrape inner bark and insert in decaying tooth; drop sap into the eyes | Pinch of bark; 1 drop 0f sap | | Once | | Has an extreme bitter sensation in the throat when sap is drop into the eyes and can cause dizziness | |
|  |  |  |  |  |  | Stem | Stomachache | Drink decoction | 1 foot length stem | | Thrice a day | |  | |
|  |  |  |  |  |  | Stem | Diabetes | Drink decoction | 1 foot length stem | | Thrice a day | |  | |
|  |  |  |  |  |  | Stem, leaf | Birth control | Drink leaf or stem decoction | 7 leaves; 1-foot length stem | | Thrice a day | |  | |
|  |  |  |  |  |  | Stem, leaf | Vomiting blood | Drink decoction | 7 leaves; 1-foot length stem | | Serve like water | |  | |
|  |  |  |  |  |  | Leaf | Headache | Apply on the forehead | 3 leaves | | Twice a day | |  | |
|  |  |  |  |  |  | Leaf | *Inaswang* | Apply on the stomach | 3 leaves | | Once a day (night) | |  | |
| *Artocarpus heterophyllus* Lam. HNUL 0021409 | Mangka | Moraceae | 0.09 | 0.08 | 0.26 | Leaf | Kidney stones | Drink decoction | 7 leaves | | Serve like water | | None | |
|  |  |  |  |  |  | Leaf | Diarrhea | Boil with *C. cainito* and *P. guajava* and drink decoction | 7 leaves | | Serve like water | |  | |
|  |  |  |  |  |  | Fruit | Amoebiasis | Eat fruit before breakfast | Medium-size sliced fruit | | Once before breakfast | |  | |
|  |  |  |  |  |  | Sap | Eczema | Apply sap on the affected area | 1 spoonful of latex | | Thrice a day | |  | |
|  |  |  |  |  |  | Bark | Stomachache | Boil and drink decoction | Handful of inner scraped bark | | Serve like water | |  | |
| *Ficus benjamina* L*.* HNUL 0021383 | Lunok | Moraceae | 0.05 | 0.05 | 0.19 | Aerial root | Postpartum care and recovery | Boil with *B. balsamifera*, *C. sumatranum*, *C. maxima*, *J. gendarussa*, and *B. spinosa* for body steaming and bathing | Handful of aerial roots | | To be performed nine days after giving birth | | None | |
|  |  |  |  |  |  | Aerial root | Stomachache, muscle pain | Pound dried aerial roots with *C. citratus* and infuse in *C. nucifera*'s oil then rub or drink | Handful of aerial roots | | Once a day (1 spoonful) | |  | |
| *Ficus septica* Burm.f. HNUL 0021326 | Lamnog/labnog | Moraceae | 0.21 | 0.21 | 0.35 | Leaf | Headache | Apply fresh or heated leaves on the forehead; apply with *B. balsamifera* on the forehead | 3 or 5 leaves; 5 or 7 leaves | | Once a day (night or afternoon); Twice a day (morning & night) | | None | |
|  |  |  |  |  |  | Leaf | Fever | Apply on the forehead | 3 or 5 leaves; 5 or 7 leaves | | Twice a day | |  | |
|  |  |  |  |  |  | Leaf | *Sinda* | Apply with *C. longa, Z. officinale*, and *J. gendarussa* on the forehead | 3 leaves | | Twice a day | |  | |
| *Moringa oleifera* Lam. HNUL 0021406 | Balunggay | Moringaceae | 0.43 | 0.36 | 0.72 | Leaf | Cough | Crush and rink extract; add extract with sugar for infants and children | 1 tablespoon or teaspoon of extract | | Twice a day | | Can cause miscarriage in pregnant woman | |
|  |  |  |  |  |  | Leaf | Tuberculosis | Crush and drink extract | 1 spoonful of extract | | Twice a day | |  | |
|  |  |  |  |  |  | Leaf | Cuts, wounds | Crush and apply extract | Handful of leaves | | Once a day | |  | |
|  |  |  |  |  |  | Leaf | Conjunctivitis | Crush and drop extract into the eyes | 3 drops of extract | | Twice a day | |  | |
|  |  |  |  |  |  | Leaf | Toothache | Chew leaves | 1 compound leaves | | Once a day | |  | |
|  |  |  |  |  |  | Leaf | UTI | Boil with *Cissus* sp. *M. charantia*, and *C. citratus* and drink decoction | Handful of leaves | | Thrice a day | |  | |
|  |  |  |  |  |  | Bark | Fracture | Apply on the affected area | 1 foot length bark | | Twice a day | |  | |
|  |  |  |  |  |  | Bark, seed | Stomachache | Drink bark decoction; eat seed | 3 or 5 seeds; handful of scraped inner bark | | Twice a day | |  | |
|  |  |  |  |  |  | Seed | Hypertension | Eat seed | 3 seeds | | Thrice a day | |  | |
| *Muntingia calabura* L. HNUL 0021364 | Sarisa | Muntingiaceae | 0.39 | 0.33 | 0.47 | Leaf, Bark | Diarrhea | Drink decoction | 7 leaves; handful of scraped inner bark | | Twice or thrice a day | | None | |
|  |  |  |  |  |  | Leaf, Bark | Stomachache | Crush and drink extract; drink bark decoction | Handful of leaves or scraped inner bark | | Half glass once a day | |  | |
|  |  |  |  |  |  | Leaf | Cough | Drink decoction | Handful of leaves | | Handful of leaves | |  | |
|  |  |  |  |  |  | Fruit | Rheumatoid arthritis | Eat fruit | Handful of fruits | | Once a day | |  | |
| *Musa balbisiana* cv. Colla HNUL 0021363 | Saging sab-a | Musaceae | 0.52 | 0.4 | 0.58 | Leaf | Dizziness | Apply on the forehead | 1 young leaf | | Twice or thrice a day | | None | |
|  |  |  |  |  |  | Leaf | Headache | Apply young leaves on the forehead or forehead and stomach | 1 young leaf | | Twice or thrice a day | |  | |
|  |  |  |  |  |  | Leaf | Fever | Apply young leaves on the forehead | 1 young leaf | | Twice a day | |  | |
|  |  |  |  |  |  | Leaf | Postpartum care and recovery | Boil dried leaves with *B. spinosa* *E. indica*, *B. balsamifer*a, *C. maxima*, *A. scholaris*, *C. sumatranum*, *C. citratus*, *C. nucifera,* and beeswax or with *I. cylindrica* or *J. gendarussa* then drink two glasses and the rest is for body steaming and bathing. | Generous amount | | To be performed nine days after giving birth | |  | |
|  |  |  |  |  |  | Stem | Cuts, wounds | Apply decaying stem on the affected area | Handful of decaying stems | | Twice a day | |  | |
| *Musa textilis* Née HNUL 0021414 | Abaka/Lanot | Musaceae | 0.04 | 0.04 | 0.08 | Shoot | Blood in feces | Drink decoction | 1 shoot | | Thrice a day | | None | |
|  |  |  |  |  |  | Shoot | Diarrhea | Grill and drink extract | 1 glass of extract | | Thrice a day | |  | |
|  |  |  |  |  |  | Stem | Stomachache | Drink water | 1 glass | | Once | |  | |
| *Musa* x *paradisiaca* L. HNUL 0021415 | Saging tundal/katsila | Musaceae | 0.04 | 0.04 | 0.08 | Fruit | Stomachache, diarrhea | Eat fruit | 1 fruit | | Thrice a day | | None | |
| *Psidium guajava* L. HNUL 0021349 | Bayabas | Myrtaceae | 0.55 | 0.4 | 0.62 | Leaf | Cuts, wounds | Crush and apply extract; boil and apply decoction as wash | Handful of leaves | | Twice or thrice a day | | None | |
|  |  |  |  |  |  | Leaf | Circumcision | Boil and apply decoction as wash | Handful of leaves | | Twice a day | |  | |
|  |  |  |  |  |  | Leaf | Vomiting blood, tuberculosis | Eat young leaves | 7 leaves | | Thrice a day | |  | |
|  |  |  |  |  |  | Leaf | Stomachache | Drink decoction; eat young leaves | 7 leaves | | Twice a day | |  | |
|  |  |  |  |  |  | Leaf | Diarrhea | Drink decoction; boil with *C. cainito* and *A. heterophyllus* then drink decoction | 7 leaves | | Serve like water | |  | |
|  |  |  |  |  |  | Leaf | Amoebiasis | Drink decoction | 7 leaves | | Serve like water | |  | |
|  |  |  |  |  |  | Leaf | Skin lesion | Boil and apply decoction as wash | 7 leaves | | Twice a day | |  | |
|  |  |  |  |  |  | Leaf | Postpartum care and recovery | Boil with *J. gendarussa*, *B.* *spinosa*, and *C. maxima* then drink one glass, and the rest is for bathing | 7 leaves | | To be performed nine days after giving birth | |  | |
| *Syzygium cumini* (L.) Skeels HNUL 0021373 | Lumboy | Myrtaceae | 0.01 | 0.01 | 0.06 | Leaf | Cough | Boil with *A. squamosa*, *C. maxima*, *A. carambola*, *A. bilimbi*, *C. microcarpa*, and C. *nucifera*'s vinegar then drink decoction | 7 leaves | | Drink as water | | None | |
| *Averrhoa bilimbi* L. HNUL 0021345 | Iba | Oxalidaceae | 0.08 | 0.07 | 0.25 | Leaf | Postpartum care and recovery | Boil with *A. carambola*, *C. maxima*, and *A. squamosa* then drink some and bath the rest | 7 leaves | | To be performed nine days after giving birth | | None | |
|  |  |  |  |  |  | Leaf, fruit | Cough | Boil alone or with *A. squamosa, C. maxima, C. microcarpa, A. carambola, T. indica, S. cumini*, and *C. nucifera*'s vinegar then drink decoction; drink fruit decoction | 7 leaves; 7 fruits | | Serve like water | |  | |
|  |  |  |  |  |  | Leaf | Kidney trouble, *doklong* | Boil with *A. bunius, S. koetjape, A. bilimbi, A. carambola* and *C. maxima* then drink and bath decoction | 7 leaves | | Serve like water | |  | |
| *Averrhoa carambola* L. HNUL 0021429 | Garangan | Oxalidaceae | 0.11 | 0.09 | 0.36 | Leaf | Typhoid fever | Boil then drink decoction and apply as sponge bath | Handful of leaves | | Thrice a day | | None | |
|  |  |  |  |  |  | Leaf | Headache | Boil with *T. indica* and bath decoction | 7 leaves | | Once a day | |  | |
|  |  |  |  |  |  | Leaf | Cough | Boil alone or with *C. maxima*, *C. microcarpa*, *T. indica*, *A. squamosa*, *A. bilimbi*, *S. cumini*, and *C. nucifera's* vinegar then drink decoction | 7 leaves | | Serve like water | |  | |
|  |  |  |  |  |  | Leaf | Kidney trouble, *doklong* | Boil with *T. indica*, *A. bunius*, *M. indica*, *A. bilimbi*, *A. carambola,* *M. maxima*, and *A. muricata* then drink and bath decoction | 7 leaves | | Serve like water | |  | |
|  |  |  |  |  |  | Leaf | Postpartum care and recovery | Boil with *C. maxima*, *J. gendarussa*, *P. carruthersii*, and *B. spinosa* or with *A. bilimbi* then drink and bath decoction | 7 leaves | | To be performed nine days after giving birth | |  | |
| *Oxalis triangularis* A.St.-Hil. HNUL 0021331 | Apat-apat | Oxalidaceae | 0.03 | 0.03 | 0.07 | Leaf | Cuts, wounds | Crush and apply | 7 leaves | | Twice a day | | None | |
| *Peperomia pellucida* (L.) Kunth HNUL 0021396 | Sinaw-sinaw | Piperaceae | 0.08 | 0.07 | 0.2 | Whole plant | Fever | Soak in warm water and drink | Handful of leaves | | Serve like water | | None | |
|  |  |  |  |  |  | Whole plant | UTI | Soak in warm water and drink | Handful of leaves | | Serve like water | |  | |
|  |  |  |  |  |  | Whole plant | Rheumatoid arthritis | Soak in warm water and drink | Handful of leaves | | Serve like water | |  | |
| *Piper betle* L. HNUL 0021314 | Buyo | Piperaceae | 0.21 | 0.16 | 0.48 | Leaf | Cough | Crush fresh or heated leaves and rub extract on the throat | 2 or 3 leaves | | Once or twice a day | | Discoloration (red) of teeth and gums | |
|  |  |  |  |  |  | Leaf | Toothache | Crush and insert in decaying tooth | Pinch of leaves | | Once | |  | |
|  |  |  |  |  |  | Leaf | Stomachache, gas pain | Wrap a pinch of *A. catechu* nut in a *P. betle* leaf daubed with slaked lime (*apog*) then chew or pound and rub extract on the stomach | 1 leaf | | Once | |  | |
|  |  |  |  |  |  | Leaf | Mumps | Apply on the affected area | 3 leaves | | Once a day | |  | |
|  |  |  |  |  |  | Leaf | Migraine | Apply on the forehead | 5 leaves | | Once a day | |  | |
|  |  |  |  |  |  | Leaf | Fever, headache | Crush heated leaves and rub extract on the head; apply on the forehead | 5 leaves | | Once a day | |  | |
|  |  |  |  |  |  | Leaf | Muscle pain | Crush and rub extract on the body | 5 leaves | | Once a day | |  | |
|  |  |  |  |  |  | Leaf | *Sinda* | Pound with *A. calamus*, *J. gendarussa*, *V. unguiculata*, *Z. officinale*, *A. sativum*, and *C. longa* and then rub extract on the body | 3 leaves | | Twice a day | |  | |
| *Antidesma bunius* (L.) Spreng HNUL 0021346 | Bugnay | Phyllanthaceae | 0.11 | 0.08 | 0.31 | Leaf | Chicken pox, measles | Boil with *C. maxima* and bath decoction; Gently strike three branches of leaves on the body then hung in the kitchen until it dries up then boil and bath decoction | 20 leaves for decoction; 3 branches of leaves for striking | | Once a day | | None | |
|  |  |  |  |  |  | Leaf | Stomachache | Apply on the stomach | 7 leaves | | Once a day | |  | |
|  |  |  |  |  |  | Leaf | Kidney trouble, *doklong* | Boil with *S. koetjape*, *M. indica*, A*. muricata,* *A. bilimbi*, *A. carambola*, and *C. maxima then* drink decoction | 7 leaves | | Serve like water | |  | |
|  |  |  |  |  |  | Leaf | Postpartum care and recovery | Boil with *B*. spinosa, *I. cylindrica*, *C. maxima*, and *J. gendarussa* or with *A. squamosa* then drink 1 glass and bath the rest | 7 leaves | | To be performed nine days after birth | |  | |
| *Bambusa spinosa* Roxb. HNUL 0021367 | Kawayan | Poaceae | 0.2 | 0.19 | 0.32 | Leaf, root | Postpartum care and recovery | Boil leaves with *B. balsamifera*, *C. maxima*, *C. sumatranum*, *J. gendarussa*, *A. scholaris*, *C.* *citratus*, *M. balbisiana* cv., *E. indica*, *C. nucifera,* and beeswax then drink two glasses of decoction and the rest is for body steaming and bathing; boil with *E. indica, O. sativa, S. elliptica* or with *B. balsamifera* then drink one glass and the rest is for body steaming and bathing; boil *I. cylindrica, A. bunius, C. maxima, J. gendarussa,* or with *D. triflorum* or *Z. officinale* or *A. carambola* then drink one glass and the rest is for body steaming and bathing | Handful of leaves | | To be performed nine days after giving birth | | None | |
|  |  |  |  |  |  | Leaf | Cancer | Boil with *E. indica* and *I. cylindrica* then drink decoction | Handful of leaves | | Serve like water | |  | |
|  |  |  |  |  |  | Leaf | UTI, kidney stones | Boil young leaves with *D. triflorum* and *I. cylindrica* and drink decoction | Handful of leaves | | Serve like water | |  | |
| *Chrysopogon aciculatus* (Retz.) Trin. HNUL 0021391 | Bariri | Poaceae | 0.04 | 0.04 | 0.13 | Root | Stomachache | Drink decoction | Handful of leaves | | Serve like water | | None | |
|  |  |  |  |  |  | Root | Tooth decay | Pound and insert in the decaying tooth | Pinch of root | | Once a day | |  | |
|  |  |  |  |  |  | Stem | Wart | Pierce the stem in the wart until it dries up | 1 stem | | Once a day | |  | |
| *Cymbopogon citratus* (DC.) Stapf HNUL 0021307 | Tanglad | Poaceae | 0.25 | 0.21 | 0.53 | Bulb | UTI | Boil alone or with *M. charantia*, *M. oleifera*, and *Cissus* sp. then drink decoction | 5 bulbs | | Serve like water | | None | |
|  |  |  |  |  |  | Bulb | Toothache/tooth decay | Crush and insert in the decaying tooth | 1 bulb | | Once | |  | |
|  |  |  |  |  |  | Bulb | Spasm | Pound with *Z. officinale* and salt with small amount of kerosene and apply on the affected area | 3 bulbs | | Once a day | |  | |
|  |  |  |  |  |  | Bulb | Stomachache | Drink decoction | 5 bulbs | | Thrice a day | |  | |
|  |  |  |  |  |  | Leaf | Muscle pain | Crush leaves with *B. balsamifera* and rub extract on the affected area | 7 leaves | | Once (night) or twice a day | |  | |
|  |  |  |  |  |  | Leaf | Limb pain | Boil with *C. utan* and apply decoction after massage | Handful of leaves | | Once | |  | |
|  |  |  |  |  |  | Leaf, root | Hypertension | Boil leaf and drink decoction; boil leaf and root and bath decoction; pound leaf and root and rub extract on the head | Handful of leaves or roots | | Drink decoction thrice a day; bath decoction once a day; rub extract once a day | |  | |
|  |  |  |  |  |  | Leaf | Rheumatoid arthritis, diabetes | Drink decoction | Handful of leaves | | Thrice a day | |  | |
|  |  |  |  |  |  | Whole plant | Halitosis | Boil and drink decoction or use decoction as mouthwash | 3 whole plants | | Twice or thrice a day | |  | |
|  |  |  |  |  |  | Leaf | Postpartum care and recovery | Boil alone or with *B. balsamifera*, *C. maxima*, *B. spinosa*, *C. sumatranum*, *A. scholaris*, *M. balbisiana* cv*.*, *E. indica*, *C. nucifera* or with *J. gendarussa*, *I. cylindrica,* and beeswax then drink two glasses and the rest is for body steaming and bathing | 7 leaves | | To be performed nine days after giving birth | |  | |
| *Eleusine indica* (L.) Gaertn. HNUL 0021354 | Plagtiki/ Bilang-bilang | Poaceae | 0.37 | 0.29 | 0.56 | Whole plant, leaf | Cancer | Boil alone or with *B. spinosa*, and *I. cylindrica* then drink decoction | Handful of leaves or whole plant | | Thrice a day | | None | |
|  |  |  |  |  |  | Whole plant | Postpartum care and recovery | Boil alone or with *C. maxima, B. spinosa*, *C. sumatranum*, *A. scholaris*, *C. citratus*, *M. balbisiana* cv*.*, *E. indica*, *C. nucifera* or with *O. sativa*, *S. elliptica*, *I. cylindrica,* and beeswax then drink two glasses and the rest is for body steaming and bathing | Handful of plants | | To be performed nine days after giving birth | |  | |
|  |  |  |  |  |  | Root, leaf | UTI | Drink root decoction; eat leaves | Handful of roots; 7 leaves | | Thrice a day | |  | |
|  |  |  |  |  |  | Stem | Vomiting blood | Eat young stem | 7 stems | | Thrice a day | |  | |
|  |  |  |  |  |  | Whole plant | Kidney stones | Drink root decoction | Handful of plants | | Serve like water | |  | |
|  |  |  |  |  |  | Root, leaf | Stomachache | Boil root or leaf alone or with *C. mindorensis* and drink decoction | 1 whole plant | | Thrice a day | |  | |
|  |  |  |  |  |  | Root, leaf | Diabetes | Boil root and leaf and drink decoction | 1 whole plant | | Serve like water | |  | |
|  |  |  |  |  |  | Root | Cough | Drink decoction | 1 whole plant | | Thrice a day | |  | |
|  |  |  |  |  |  | Whole plant | Diarrhea | Drink decoction | 5 whole plants | | Twice a day | |  | |
| *Imperata cylindrica* (L.) Raeusch. HNUL 0021304 | Kogon | Poaceae | 0.25 | 0.24 | 0.46 | Shoot, root | Stomachache | Boil root or shoot and drink decoction | Roots of 5 plants; 5 shoots | | Thrice a day | | None | |
|  |  |  |  |  |  | Shoot, root | UTI | Boil root with *C. asiatica* and drink decoction; boil shoot alone or with *D. triflorum* and *B. spinosa* then drink decoction | Roots of 5 plants; 5 shoots | | Thrice a day or serve like water | |  | |
|  |  |  |  |  |  | Shoot | Postpartum care and recovery | Boil with *M. balbisiana* cv*.*, *J. gendarussa*, *Z. officinale*, and sugar then drink decoction; boil with *J. gendarussa*, *A. squamosa*, *C. maxima*, and *A. bunius* then drink one glass; boil with *J. gendarussa*, *C. citratus*, and *B. spinosa* for body steaming; boil with *B. balsamifera*, *B. spinosa*, and *C. maxima* then bath decoction; boil with *B. spinosa, S. elliptica* and *E. indica* then bath decoction; boil with *C. citratus, B. spinosa, C. maxima, B. balsamifera*, and *M. balbisiana* cv*.* then bath decoction; boil with *B. spinosa*, *A. bunius, C. maxima*, and *J. gendarussa* then drink one glass and bath the rest | 7 shoots | | To be performed nine days after giving birth | |  | |
|  |  |  |  |  |  | Shoot | Cancer | Boil with *E. indica* and *B. spinosa* then drink decoction | 7 shoots | | 1 week | |  | |
|  |  |  |  |  |  | Shoot | Kidney stones | Boil alone or with *D. triflorum* and *B. spinosa* then drink decoction | Handful of shoots | | Thrice a day or serve like water | |  | |
|  |  |  |  |  |  | Shoot | Headache | Drink decoction | Handful of shoots | | Drink as water | |  | |
|  |  |  |  |  |  | Root | Diarrhea | Drink decoction | 7 whole plant's roots | | Twice a day | |  | |
| *Oryza sativa* L. HNUL 0021369 | Paray | Poaceae | 0.13 | 0.08 | 0.22 | Root | Postpartum care and recovery | Boil alone or with *E. indica*, *B*. *spinosa*, and *S. elliptica* drink some of the decoction then the rest is for body steaming and bathing. | Handful of roots | | To be performed nine days after giving birth | | None | |
|  |  |  |  |  |  | Seed | Bloated stomach/gas pain | Chew with *A. sativum* and apply on the stomach; chew with *Z. officinale* and *V. unguiculata* and apply on the stomach; mix with *N. tabacum* and apply on the stomach | Pinch | | Twice a day | |  | |
|  |  |  |  |  |  | Seed | Animal (dog) bite | Chew and apply on the bitten area | Pinch | | Thrice a day | |  | |
| *Saccharum officinarum* L. HNUL 0021385 | Tubo | Poaceae | 0.03 | 0.03 | 0.07 | Stem | Hoarseness | Remove outer skin and leave overnight then chew and drink extract in the morning | At least 1-foot-long stem | | Once | | None | |
| *Zea mays* L. HNUL 0021389 | Mais | Poaceae | 0.05 | 0.04 | 0.08 | Flower | UTI | Boil silk with *M. pudica* and drink decoction | Handful of silk | | Serve like water | | None | |
|  |  |  |  |  |  | Flower | Kidney stone | Drink silk decoction | Handful of silk | | Serve like water | |  | |
| *Chrysophyllum cainito* L. HNUL 0021348 | Star apol | Sapotaceae | 0.35 | 0.32 | 0.36 | Leaf, bark | Diarrhea | Drink decoction of leaves or bark; boil alone or with *P. guajava* and *A. heterophyllus* and drink decoction | 7 leaves; handful of scraped bark | | Thrice a day | | None | |
|  |  |  |  |  |  | Bark, root | Stomachache | Boil roots or bark alone or with *P. americana* and drink decoction | Handful of scraped inner bark or sliced roots | | Drink as water | |  | |
| *Capsicum annuum* L. HNUL 0021378 | Katumbal | Solanaceae | 0.13 | 0.12 | 0.44 | Leaf | Fever | Drink decoction | Handful of leaves | | Thrice a day | | None | |
|  |  |  |  |  |  | Leaf | Typhoid fever | Crush heated leaves and rub on the head | Handful of leaves | | Once a day | |  | |
|  |  |  |  |  |  | Leaf | Headache | *To-om* then crush and rub extract on the body | Handful of leaves | | Once a day | |  | |
|  |  |  |  |  |  | Leaf | Caterpillar dermatitis | Crush with a pinch of salt and rub extract on the affected area | Handful of leaves | | Once | |  | |
|  |  |  |  |  |  | Leaf | Skin lesion | Crush and rub extract on the affected area | Handful of leaves | | Once a day | |  | |
|  |  |  |  |  |  | Root, fruit | Hypertension | Drink decoction | Handful of leaves | | Serve like water | |  | |
|  |  |  |  |  |  | Leaf | *Sinda* | Apply with *C. longa* and *Z. officinale* on the forehead and stomach | 7 leaves | | Twice a day | |  | |
| *Solanum lycopersicum* L. HNUL 0021387 | Kamatis | Solanaceae | 0.04 | 0.04 | 0.08 | Leaf | Bloated stomacarbh | Crush and rub extract on the stomach; Apply heated leaves on the stomach | Handful of leaves | | Twice a day | | None | |
|  |  |  |  |  |  | Leaf | Diarrhea | Apply heated leaves on the stomach | Handful of leaves | | Twice a day | |  | |
| *Nicotiana tabacum* L. HNUL 0021398 | Tabako | Solanaceae | 0.08 | 0.03 | 0.07 | Leaf | Stomachache; bloated stomach | Pound used cigar (*N. tabacum*) head with *O. sativa* and apply on the stomach | I cigar head | | Twice a day | | None | |
| *Solanum melongena* L. HNUL 0021430 | Tarong | Solanaceae | 0.01 | 0.01 | 0.06 | Leaf | Stomachache | Apply on the stomach | 2 or 3 leaves | | Once | |  | |
| *Nauclea orientalis* (L.) L. HNUL 0021343 | Bangkal | Rubiaceae | 0.01 | 0.01 | 0.06 | Leaf | Headache | Apply on the forehead | 1 leaf | | Twice a day | | None | |
| *Morinda citrifolia* L. HNUL 0021351 | Anino | Rubiaceae | 0.17 | 0.08 | 0.35 | Fruit | Cancer | Drink decoction | 3 fruits | | Thrice a day | | None | |
|  |  |  |  |  |  | Fruit, leaf | Diabetes | Drink leaf or root decoction | 3 fruits; 3 leaves | | Thrice a day | |  | |
|  |  |  |  |  |  | Fruit, leaf | UTI | Drink fruit decoction; apply leaves on the stomach area | 3 fruits; 3 leaves | | Thrice a day | |  | |
|  |  |  |  |  |  | Fruit | Kidney problems | Drink decoction | 3 fruits | | Thrice a day | |  | |
|  |  |  |  |  |  | Fruit, leaf, bark | Rheumatoid arthritis | Drink fruit, leaf or bark decoction | 3 fruits; 7 leaves; handful of scaped inner bark | | Thrice a day or serve like water | |  | |
|  |  |  |  |  |  | Fruit, leaf | Hypertension | Drink fruit or leaf decoction | 3 fruits; 7 leaves | | Serve like water | |  | |
|  |  |  |  |  |  | Fruit, root | *Doklong* | Drink fruit and root decoction | 3 fruits, palm-length root sliced | | Serve like water | |  | |
| *Mussaenda philippica* A.Rich. HNUL 0021405 | Lakboy | Rubiaceae | 0.03 | 0.03 | 0.07 | Leaf | Headache, dizziness | Apply on the forehead | 3 leaves | | Once a day | | None | |
| *Citrus maxima* (Burm.) Merr. HNUL 0021347 | Kabugaw | Rutaceae | 0.29 | 0.2 | 0.47 | Leaf | Fever | Boil with *A. bilimbi* and *A. squamosa* and apply as sponge bath; boil and bath decoction; | 7 leaves | | Once a day | | None | |
|  |  |  |  |  |  | Leaf | Cough | Boil with *A. bilimbi* and *A. squamosa* and apply as sponge bath; Boil with *A. carambola, A. bilimbi, T. indica, C. microcarpa, S. cumini, S. koetjape,* and *C*. *nucifera*'s vinegar then drink decoction | 7 leaves | | Thrice a day | |  | |
|  |  |  |  |  |  | Leaf | *Doklong* | Drink decoction; Boil with *A. carambola*, *A. bilimbi*, *T. indica*, *C. microcarpa*, *S. cumini*, S. *koetjape*, and *C. nucifera*'s vinegar then drink decoction | 7 leaves | | Serve like water | |  | |
|  |  |  |  |  |  | Leaf | Kidney trouble | Boil with *S. koetjape*, *A. bunius*, *M. indica*, *A. muricata*, *C. bilimbi,* and *A. carambola* then drink and bath decoction | 7 leaves | | Serve like water | |  | |
|  |  |  |  |  |  | Leaf | Postpartum care and recovery | Boil with *B. balsamifera*, *B. spinosa,* *A. scholaris*, *C. sumatranum*, *C. citratus*, *M. balbisiana* cv*.*, *E. indica*, *C. nucifera*, and beeswax then drink two glasses and bath the rest; boil with *B. balsamifera*, *F. benjamina, C. sumatranum, B. spinosa,* and *J. gendarussa* for body steaming and bathing; boil with *I. cynlindrica, A. bunius, B. spinosa*, and *J. gendarussa* then drink one glass and bath the rest; boil with *A. squamosa, A. bilimbi*, and *A. carambola* or with *P. carruthersii* or *P. guajava* then drink and bath decoction | 7 leaves | | To be performed nine days after giving birth | |  | |
|  |  |  |  |  |  | Leaf | Chicken pox | Boil with *A. bunius* and bath decoction | 7 leaves | | Once a day | |  | |
|  |  |  |  |  |  | Leaf | *Inaswang* | Apply with *C. sappan*, *B. balsamifera*, and *J. gendarussa* on the stomach | 7 leaves | | Once | |  | |
| *Citrus microcarpa* Bunge HNUL 0021412 | Suha | Rutaceae | 0.07 | 0.07 | 0.16 | Fruit, leaf | Cough | Drink juice; Boil leaf with *A. squamosa*, *C. maxima*, *A. carambola*, *A. bilimbi*, *S. cumini*, and *S. koetjape* then drink decoction | 7 leaves | | Serve like water | | None | |
|  |  |  |  |  |  | Leaf | *Doklong* | Boil with *C. maxima*, *A. carambola*, *A. bilimbi*, T. indica, *S. koetjape*, *S. cumini*, and *C. nucifera*'s vinegar then drink decoction |  | |  | |  | |
| *Pipturus arborescens* (Link) C.B. Rob*.* HNUL 0021336 | Hiniramay | Urticaceae | 0.05 | 0.05 | 0.19 | Sap | Boils | Apply sap on the affected area | Generous amount sap | | Thrice a day | | None | |
|  |  |  |  |  |  | Bark | *Hiwit* | Scrape inner bark and mix with *Amomum* sp. then crush and drink extract | Handful of scraped bark | | Once a day | |  | |
|  |  |  |  |  |  | Bark | Breast engorgement | Scrape inner bark and apply on the breast | Handful of scraped bark | | Once a day | |  | |
|  |  |  |  |  |  | Bark | Shingles | Scrape inner bark and apply | Handful of scraped bark | | Once a day | |  | |
| *Stachytarpheta jamaicensis* (L.) HNUL 0021344 | Sentimento | Verbenaceae | 0.15 | 0.15 | 0.19 | Leaf | Cuts/wounds | Crush and apply extract on the affected area | Handful of leaves | | Once a day | | None | |
| *Cissus* sp. HNUL 0021328 | Ginseng | Vitaceae | 0.05 | 0.03 | 0.12 | Aerial root | UTI | Boil with *M. charantia*, *M. oleifera,* and *C. citratus* then drink decoction | Handful of aerial roots | | Thrice a day | | None | |
|  |  |  |  |  |  | Aerial root | Headache | Boil with *Z. officinale, C. longa*, and *C. citratus* then drink decoction | Handful of aerial roots | | Twice a day | |  | |
| *Leea guineensis* G. Don HNUL 0021334 | Mamali | Vitaceae | 0.01 | 0.01 | 0.06 | Leaf | *Kolebra* | Burn and add ash in water then drink | 7 leaves | | Once a day | | None | |
| *Tetrastigma* sp. Planch. HNUL 0021386 | Alumperan | Vitaceae | 0.03 | 0.03 | 0.12 | Leaf | Cuts/wounds | *To-om* and apply | Handful of leaves | | Thrice a day | | None | |
|  |  |  |  |  |  | Leaf | Lower back pain | Apply directly on the affected area | Handful of leaves | | Twice a day | |  | |
| *Alpinia galanga* (L.) Willd. HNUL 0021350 | Langkawas | Zingiberaceae | 0.05 | 0.05 | 0.1 | Rhizome | Pityriasis versicolor | Pound rub extract, rub on the affected area | Palm-length rhizome | | Once a day | | None | |
| *Amomum* sp. HNUL 0021342 | Tugis | Zingiberaceae | 0.07 | 0.05 | 0.19 | Shoot | Headache | Pound alone or with *E. philippinensis*, *C. speciosus*, *L. circinnatum*, *C. longa*, and *O. officinale* then rub extract on the head | 1 shoot | | Once a day | | None | |
|  |  |  |  |  |  | Stem | *Hiwit* | Crush with *P. arborescens* and drink extract; pound and rub extract | 3 stems | | Once a day | |  | |
|  |  |  |  |  |  | Stem | Hypertension | Pound and rub extract | 3 stems | | Once a day | |  | |
| *Curcuma longa* L. HNUL 0021312 | Kalawag | Zingiberaceae | 0.79 | 0.47 | 0.87 | Rhizome | *Sinda* | Pound and rub extract on the head; slice rhizome and apply on the forehead with *Z. officinale, P. carruthersii, J. gendarussa*, and *T. crispa*; apply with *C. annuum* and *Z. officinale* in the forehead and stomach; pound with *Z. officinale* and rub extract on the body | 7 thinly sliced rhizomes | | Twice a day | | None | |
|  |  |  |  |  |  | Rhizome | Headache, fever | Pound and rub extract on the head; slice rhizome and apply with *P. carruthersii*, and *Z. officinale* or with *J. gendarussa* on the forehead; pound and add sugar in extract then drink; pound with *C. sappan* and *Z. officinale* then drink some extracts and rub on pulse points; pound with *A. calamus* and rub extract on the body; Pound with *E. philippinensis, C. speciosus, L. circinnatum, Amomum* sp., and *Z. officinale* and rub extract on the head; boil with *Cissus* sp*., Z. officinale*, and *C. citratus* then drink decoction | 7 thinly sliced rhizomes | | Twice a day | |  | |
|  |  |  |  |  |  | Rhizome | Muscle pain | Pound and rub extract on the body | 1 knob | | Once a day | |  | |
|  |  |  |  |  |  | Rhizome | Stomachache | Drink decoction | 3 knobs | | Twice a day | |  | |
|  |  |  |  |  |  | Rhizome | Typhus | Pound then add *C. nucifera'*s vinegar and rub on the head | 5 knobs | | Twice a day | |  | |
|  |  |  |  |  |  | Rhizome | Typhoid fever | Pound and rub extract on the head; pound with *Z. officinale* and *C. nucifera*'s vinegar or with *E. philippinensis* then rub extract on the head and stomach | 3 knobs | | Thrice a day | |  | |
|  |  |  |  |  |  | Rhizome | Memory loss, cancer | Drink decoction | 7 knobs | | Thrice a day | |  | |
|  |  |  |  |  |  | Rhizome | Bloated stomach | Apply with *B. balsamifera* and *J. gendarussa* on the stomach | 7 sliced rhizomes | | Once a day | |  | |
|  |  |  |  |  |  | Rhizome | Cuts, wounds, tetanus | Pound and apply | 1 knob | | Once | |  | |
|  |  |  |  |  |  | Rhizome | Tooth decay | Insert in the decaying tooth | A pinch of rhizome | Once | |  | |  |
| *Etlingera philippinensis* (Ridl.) R.M.Sm. HNUL 0021323 | Tagbak | Zingiberaceae | 0.12 | 0.12 | 0.21 | Stem | Typhoid fever | Pound shoot and apply extract on the head; pound with *C. longa, Z. officinale*, and *C*. *nucifera*'s vinegar then rub extract on the head and stomach and apply on the forehead | 1 stem | Thrice a day | | None | |  |
|  |  |  |  |  |  | Stem | Tuberculosis | Eat basal stem core | 1 stem | Once a day | |  | |  |
|  |  |  |  |  |  | Stem | Headache | Pound and apply on the forehead; pound with *C. speciosus*, *Amomum* sp., *L. circinnatum*, *C. longa*, and *Z. officinale* then rub extract | 1 stem | Thrice a day | |  | |  |
| *Kaempferia galanga* L. HNUL 0021376 | Kusol | Zingiberaceae | 0.04 | 0.04 | 0.12 | Rhizome | Boils | Apply on the affected area | 1 thin sliced rhizome | Twice a day | | None | |  |
|  |  |  |  |  |  | Rhizome | Mumps | Apply on the affected area | 5 thin slice rhizomes | Twice a day | |  | |  |
|  |  |  |  |  |  | Rhizome | Wounds | Apply on the affected area | 1 thin sliced rhizome | Twice a day | |  | |  |
| *Zingiber officinale* Roscoe HNUL 0021341 | Luy-a | Zingiberaceae | 0.49 | 0.43 | 0.78 | Rhizome | *Sinda* | Apply slice rhizome on the forehead with *C. longa*, *P. carruthersi*i, *and J. gendarussa*, or with *T. crispa*; pound with *C. longa* and rub extract on the body; Apply with *C. annuum* and *C. longa* on the forehead and stomach; Pound with *A. calamus*, *J. gendarussa*, *P. betle*, *A. sativum*, *V. unguiculata,* and C. *longa* and rub extract on the body, Apply with *F. septica,* *J. gendarussa*, and *C. longa* on the forehead | 7 thinly sliced rhizomes | Thrice a day | | None | |  |
|  |  |  |  |  |  | Rhizome | Headache | Apply sliced rhizome with *P.* *carruthersii* and *C. longa* or with *J. gendarussa*, or *B. balsamifera* on the forehead; Pound with *E. philippinensis*, *Amomum* sp., *C. speciosus*, *L. circinnatum*, *C. longa*, and *Z. officinale* and rub extract on the head; boil with *Cissus* sp., *C. longa, C. citratus* and drink decoction; Pound grilled rhizome and rub extract on the body | 7 thinly sliced rhizomes | | Thrice a day | |  | |
|  |  |  |  |  |  | Rhizome | Bloated stomach, stomachache | Chew with *V. unguiculata* and *O. sativa* grain and rub on the stomach; Slice and apply with *B. balsamifera*, and *J. gendarussa* apply on the stomach; drink decoction | 1 small knob | | Twice a day | |  | |
|  |  |  |  |  |  | Rhizome | Typhoid fever | Pound with *C. longa* and *C. nucifera* (vinegar) and rub extract on the head and stomach; pound grilled rhizome and add some alcohol then rub on the body; pound with *E. philippinensis, C. longa*, and *C. nucifera*'s vinegar then rub extract on the head and stomach and apply on the forehead | 3 knobs | | Thrice a day | |  | |
|  |  |  |  |  |  | Rhizome | Fever | Apply with *C. longa* or with *J. gendarussa* and *P. carruthersii* on the forehead | 3 knobs | | Thrice a day | |  |  |
|  |  |  |  |  |  | Rhizome | Spasm | Pound with *C. citratus*, salt with small amount of kerosene and rub extract on the affected area; heat and apply | 3 knobs | | Twice a day | |  |  |
|  |  |  |  |  |  | Rhizome | Tonsilitis | Drink decoction | 5 knobs | | Thrice a day | |  |  |
|  |  |  |  |  |  | Rhizome | Tetanus | Heat and apply | 1 sliced rhizome | | Thrice a day | |  |  |
|  |  |  |  |  |  | Rhizome | Hoarseness | Chew and drink extract | 1 small knob | | Thrice a day | |  |  |
|  |  |  |  |  |  | Rhizome | Hearing impairment | Heat and apply on the back of the ear | 1 thin slice rhizome | | Twice a day | |  |  |
|  |  |  |  |  |  | Rhizome | Postpartum care and recovery | Boil with *C. sumatranum*, *J. gendarussa*, *B. balsamifera*, *B. spinosa*, *C. citratus*, and *Z. officinale* then bath decoction; boil with *J. gendarussa*, *M. balbisiana* cv., and *I. cylindrica* then drink decoction | 7 sliced rhizomes | | To be performed nine days after giving birth | |  |  |
|  |  |  |  |  |  | Rhizome | Chill | Pound and rub extract on the body | 3 knobs | | Twice a day | |  |  |

^a^*Inaswang*: a condition that is culturally believed that caused by *aswang* (witch), *aswang* is a shapeshifting creature that eat human body parts such as liver and heart; *sinda*: caused by the charm of spirits or supernatural beings with symptoms like dizziness or fever*; hiwit*: "sorcery" makes body organs deteriorates; *kolebra*: with symptoms of severe stomachache, nausea, chills, shortness of breath, and paleness; *doklong*: similar to "relapse" and sometimes accompanied with other symptoms like headache, muscle pain, and weakness.

^b^*To-om*: a preparation in which a handful of leaves is divided in two halves, one half is wrapped in *Musa* sp. leaf then grilled or heated. The other half is crushed with a pinch of salt. Combine the two halves then crush and rub the extracts on the body.
