# Supplementary material for: Ethnobotanical Documentation of Medicinal Plants Used by the Indigenous Panay Bukidnon in Lambunao, Iloilo, Philippines
Source: Front Pharmacol. 2022 Jan 10;12:790567. doi: 10.3389/fphar.2021.790567 (PMC8784692; doi:10.3389/fphar.2021.790567)
Supplement: Supplementary file 3 [file DataSheet1.pdf]

Supplementary Material: Questionnaire

Ethnobotanical Documentation of the Medicinal Plants Used by the *Panay Bukidnon* in Brgy. Caguisanan, Lambunao, Iloilo, Philippines

Part 1: Personal Profile

Apelyido (Surname*):* ____________________ *Ngaran* (First Name): ___________
Pagkatawo (Sex): _____ *Lalaki* (Male) ____ *Babaye* (Female)
Edad (Age): ________ *Edukasyon* (Education): ______________
Estado Sibil (Civil Status): __*May asawa* (Married) __ *Waay Asawa* (Single) ___*Balo* (Widowed)
*Puloy-an (*Address): ______________ *Trabaho* (Occupation): ______________

Part 2: Health Problem and Medicinal Plants Used

1. *Ano nga mga masakit sa lawas ang inyo naagyan o nabatyagan?*
   What are the health problems have you experienced?

2. *Ano nga (mga) tanom ang inyo ginagamit sa pagbulong sa mga masakit nga inyo nabatyagan?*
   What is/are the plant/s you use to treat your health problems/s?

3. *Parte ka tanom nga ginagamit sa pagpaayad ka mga sakit.* Plant part/s used to treat the health problems.
   a. *Panit* (bark)
   b. *Sanga* (stem)
   c. *Gamot* (root)
   d. *Pul-o* (shoot)
   e. *Liso* (seed)
   f. *Dahon* (leaf)
   g. *Prutas* (fruit)
   h. *Bukol* (bud)
   i. *Bulak* (flower)
   j. *Dugos* (latex or sap)
   k. *Ibabaw nga parte* (aerial parts)
   l. *Iban pa* (others)

4. *Porma kang bulong.* Administration forms.
   a. *Ginakumos o lubak* (crush/pound)
   b. *Ginala-ga* (decoction)
   c. *Ginaka-on* (eat)
   d. *Ginahuruman sa tubig* (soak in water)
   e. *Gina-inom* (drink)
   f. *Sunog para sa aso* (burn as incense)
   g. *Ginahampol diretso* (apply directly)
   h. Ginakagod (scrape)
   i. *Parigos o sibin* (bath or sponge bath)
   j. *Ginapaturo* (drop)

5. *Karakuon ka bulong*. (Quantity or dosage of the medicine.)

6. *Malain nga epekto ka tanom nga buong*. Adverse or side effects of the plant/s.

7. *Lugar kung diin ginabu-ol ang mga tanom?* (Where do you get the plant/s you used?)
   a. *Ginapatubo sa palibot kang balay* (Cultivated around the house*)*
   b. *Sa pihak nga baryo* (From the neighboring barangay)
   c. *Sa kakahuyan* (forest/wild)
   d. *Sa iban pa nga lugar* (other places)____________

Tabulated form of the questions for health problem/s and medicinal plants used.

Ngaran ka Tanom<br><br>Plant Name	*Parte ka tanom*<br><br>Plant part/s used	*Sakit*<br><br>Disease/sickness	*Porma sa pagbulong*<br><br>Form of administration	*Karakuon ka bulong*<br><br>Quantity or dosage of the medicine	*Malain nga epekto ka tanom nga ginabulong*<br>Adverse or side effects of plants	*Lugar kung diin ginabu-ol ang mga tanom*<br>Where do you get the plant/s you used
Ex. Kalawag	*Sulod*	*Sakit ulo*	*Bukbokon kag banyos ang duga sa ulo*	*Pito ka kihad; banyos duha ka beses sa isa ka adlaw (aga kag gab-i)*	*Waay*	*Tanom sa likod balay*
Curcuma longa L.	Rhizome	Headache	Pound and rub extract on the head	Seven sliced rhizomes; Rub extract twice a day (morning and night)	None	Planted in the backyard
